# Assessing Factors that Influence Psychiatric Referral Time and Postpartum Follow-Up in Obstetric Patients

**DOI:** 10.1007/s10597-026-01608-7

**Published:** 2026-04-30

**Authors:** Lauren Womer, Derek Harmanli, Landon Leininger, Hannah McBride, Shayan Jalali, Sarah Tabi

**Affiliations:** 1 Temple University, Philadelphia, USA; 2 St. Elizabeth’s Medical Center Psychiatry Residency, Boston, USA; 3 Drexel University, Science and Health Systems, Philadelphia, USA

**Keywords:** Obstetric psychiatry, Postpartum follow up, Psychiatric referral, Peripartum psychiatry

## Abstract

There is a clear shortage of psychiatric care in the population of peripartum obstetric patients in the United States(Kelly et al., 2001; Claridad, 2025). There is variability in both screening for mental illness and availability of psychiatric care which leads to a substantial gap in healthcare for such patients, particularly non-white patients. Additionally, during the postpartum period, the attendance rates to medical appointments are affected by race, ethnicity and socioeconomic status, further perpetuating the aforementioned healthcare disparity(Wilcox et al., 2016). The key purpose of this study was to understand if and how rates of seeing a reproductive psychiatrist are affected by psychiatric referral time (prenatally versus postnatally) and, if and how seeing a reproductive psychiatrist affect postpartum healthcare follow in obstetric patients. Patients referred to the reproductive psychiatrist between January to December of 2023 were chart reviewed in EPIC and data was recorded in RedCap (N=71). Data recorded included: age, ethnicity, education, employment, marital status, substance use, and medical factors such as psychotropic medication use and psychiatric diagnoses. Separate Chi Squared tests were employed to evaluate two variables including: referral time (before delivery versus after delivery versus unknown) and if patients were lost to follow up after delivery (yes versus no), respectively. There were 71 obstetric patients evaluated with a mean age of 28 and an age range of 17-39. Most were Black (N=31) or Hispanic/ Latino (N=24). The results indicate that patients referred prenatally had 3.26 times higher odds of meeting with the reproductive psychiatrist (N = 44, 62%) compared to those referred postnatally (p = 0.0002). Patients with a history of substance use, marijuana use, presence of long-term romantic partners, use of prescribed psychotropic medications, history of any bipolar disorder, and had their diagnosis made by the reproductive psychiatrist were associated with higher rates of being referred to psychiatry prenatally, while patients diagnosed with postpartum depression were associated with higher rates of being referred postnatally. The odds of patients who met with the reproductive psychiatrist (N=43, 61.4%) being lost to postpartum healthcare follow up were 3.63 times lower compared to the odds of patients who did not meet with the psychiatrist (p=0.012). Furthermore, patients who used alcohol, had a diagnosis of postpartum depression, had their mental health conditions diagnosed by the reproductive psychiatrist, were taking a prescribed psychotropic medication, particularly antidepressants including sertraline and escitalopram, were associated with lower rates of being lost to postpartum healthcare follow up. Patients who were unemployed were associated with higher rates being lost to healthcare follow up. This study explores how timing of psychiatric referral in obstetric patients affects postpartum healthcare appointment attendance, highlighting the role of early psychiatric intervention in potentially improving postpartum healthcare engagement. Knowledge of these findings may encourage providers to consider psychiatric referral more readily. Similarly, by understanding individual factors that independently influence psychiatric referral time and healthcare follow up rates, providers can bring awareness to what impacts their decision, or lack thereof, to refer patients to psychiatry.

**Supplementary Information:**

The online version contains supplementary material available at https://doi.org/10.1007/s10597-026-01608-7.

## Introduction

A common theme in obstetric patients, particularly patients of color, is a clear shortage of psychiatric care or consideration in the United States(Kelly et al., 2001; Claridad, 2025). Not one specific identifiable factor is responsible for this absence, but a multitude of factors that influence psychiatric screening as well as access to psychiatric care once a need is recognized. In assessing access to psychiatric care in obstetric patients, it is vital to also evaluate how such access, or lack thereof, affects postpartum follow up of these patients within the healthcare system.

Screening for psychiatric conditions such as peripartum depression is a key component in caring for obstetric patients(O’Connor et al., 2016; Sidebottom et al., 2021). By identifying potential mental illness in these patients, such as peripartum depression, providers mitigate the detrimental effects these conditions, such as increased morbidity and mortality for the patient and child(Lusskin et al., 2007). Prenatal mental health screening recommendations from the American College of Obstetrics and Gynecology (ACOG) suggest screening at the initial prenatal visit, later in pregnancy and at postpartum visits(“Screening and Diagnosis of Mental Health Conditions During Pregnancy and Postpartum,” 2023). For postpartum screening, one study says the optimal time to screen is 4–6 weeks after delivery(Sit & Wisner, 2009), although another study that evaluated 22 articles related to screening and diagnosis found that 43% screened for postpartum depression from 0 to 3 months, 19% screened up to 6 months, and 38% screened up to 12 months(Moraes et al., 2017).

While there are recommended time points to screen for peripartum depression as noted above, there is variability in execution based on the clinic site, physical location, and other factors. Differences in execution may affect screening rates. One study that assessed both prenatal and postpartum screening of 7,000 patients found that 65.1% were screened before giving birth, and 64.4% were screened in the postpartum period. The most prominent predictor of variation in screening was clinic site, with 23–30% variability in screening prevalence(Sidebottom et al., 2021). On another note, the same study noted no disparities found in prenatal screening based on race, but non-white women including African American, Asian, Native American and multi-racial were found to be screened less by providers than white women in the postpartum period(Sidebottom et al., 2021). Furthermore, providers screened women on Medicaid/Medicare less than women on private insurance(Sidebottom et al., 2021).

Variability in screening and lack of awareness of psychiatric needs in obstetric patients directly influences availability of psychiatric care to this patient population. To expand upon this, a recent study implemented a multidisciplinary consult liaison model in an obstetrics unit to identify patients at risk for or currently experiencing perinatal mental and anxiety disorders and had a consult rate of 21.7%, demonstrating the great need for psychological and psychiatric care in obstetric patients(Lebin et al., 2024). Most of these consults, 94%, were completed by a social worker or psychologist, although a psychiatrist completed consults that involved medication management, and patients with a history of anxiety or bipolar disorders(Lebin et al., 2024). In terms of race and ethnicity as it relates to psychiatric referral, another study from 2024 found that among perinatal patients with depressive symptoms, non-Hispanic white women were more likely to receive psychiatric referral than women of color(Boama-Nyarko et al., 2024). This deficiency of psychiatric referral in obstetric patient populations, particularly non-white populations, as well as the inconsistencies among peripartum mental illness screening propagate the issue of not only poor psychiatric outcomes, but overall, a probable decline in wellness and health of these patients. Furthermore, the need for psychiatric care does not end after patients give birth.

The postpartum period is critical for the long-term health of the patient; current guidance from ACOG states that the postpartum period should be viewed as a “fourth trimester” to better the outcomes of the patient and their infant(“ACOG Committee Opinion No. 736,” 2018). In a literature review from 2023, it was discovered that there was a range of 24.9% to 96.5% of postpartum visits being attended, with a mean of 72.1%(Attanasio et al., 2022). Despite this range, it is evident that a substantial proportion of obstetric patients do not attend their postpartum visits, which is a critical period to maintain the health and wellbeing of the patient and child(Attanasio et al., 2022).

In terms of who attends postpartum follow up visits, a range of socioeconomic factors come into play. Postpartum visits are where patients are most likely to be screened for postpartum depression and other postpartum mental health conditions(Blenning & Paladine, 2005). Not only does low socioeconomic status impact screening for postpartum depression as stated above, it also can lead to decreased adherence of postpartum visit attendance. To elaborate, a 2016 study found that postpartum visit non-attendance was found to be greatest in patients who were from a minority ethnic background, were younger (< 20 years old), and who relied upon state funded health insurance(Wilcox et al., 2016). This demonstrates a gap in postpartum care for women of low socioeconomic status, leading to less comprehensive treatment for mental health disorders such as postpartum depression in the setting of visit nonattendance.

As stated above, other studies have demonstrated the independent effects of psychiatric screening, referral time to psychiatry and postpartum follow up rates in the context of socioeconomic and cultural factors. The key aim of this study is to understand if and how rates of seeing a reproductive psychiatrist are affected by psychiatric referral time (prenatally versus postnatally), and if and how meeting with a reproductive psychiatrist affects postpartum healthcare follow up in obstetric patients. The secondary aim of this study is to determine which socioeconomic and cultural factors independently influence referral time and eventual postpartum healthcare follow up. By understanding these relationships, obstetric providers can use the information to guide decision making as it relates to involving reproductive psychiatry when a concern is present. Patients benefit from this if knowledge of when to refer leads to involvement of a psychiatrist who can fully gain understanding and manage mental health diagnoses of such patients. Also, this gives patients another point of contact within the healthcare system.

## Materials and Methods

A total of 71 obstetric patients in the Temple University Health System (TUHS) that were referred to a reproductive psychiatrist were chart reviewed to examine factors that influence psychiatric referral time (prenatally versus postnatally), and eventual postpartum healthcare follow up. Chart review was done through the electronic medical record used by TUHS, EPIC, and data was recorded in RedCap. All patients included were referred to psychiatry either prenatally or in the postpartum period between January to December of 2023. Patients evaluated were living in the greater Philadelphia area and had a mean age of 28 and an age range of 17–39. A total of 43.7% were Black or African American, 33.8% were Hispanic or Latino of any race, 21.1% were White or Caucasian, and 2.8% did not have ethnicity noted in the chart. Institutional Review Board (IRB) exemption was granted for this project by Temple University’s IRB on the basis that this study was conducted using only one reproductive psychiatrist’s patients.

### Key Terms and Definitions

Prenatal referral is defined as psychiatric referral any time from conception to prior to birth. Postpartum referral is defined as psychiatric referral any time after birth up to 6 months after birth. Postpartum healthcare follow up or healthcare follow up in the postpartum period is defined as follow up with any healthcare provider (obstetric, primary care, psychiatry). Lost to postpartum healthcare follow up is no healthcare follow up within 8 weeks of giving birth. Having met with the reproductive psychiatrist is defined as one or more visits with the reproductive psychiatrist.

### Variables

Analysis of the data recorded focused on how referral time and postpartum follow up related to further sociologic factors such as employment, marital status (single, married, unmarried with long-term partner, divorced, widowed, unknown), history of substance use (yes, no, unknown), education level (below high school, high school degree, associate’s degree, bachelor’s degree, graduate degree, unknown). Medical variables such as prescribed psychotropic medication use (yes, no and which medications), preexisting mental health diagnoses (yes, no and which diagnoses), and whether the patients had met with the reproductive psychiatrist once referred (yes, no) were also assessed.

### Statistical Analyses

Separate Chi Squared tests were employed to evaluate two main variables (A and B) including: (A) referral time (before delivery versus after delivery versus unknown) and (B) if patients were lost to postpartum healthcare follow up (yes versus no). For the “referral time” evaluation, or variable A, a Chi Squared test was used for 71 patients to account for three options of referral timing which include before birth, after birth and unknown. Due to the presence of “unknown” as a possibility for variable A, separate analyses were used for variables A and B. In terms of the “lost to healthcare follow up” evaluation, or variable B, a Chi Squared test was used for 70 patients with two possible outcomes: lost to healthcare follow up or having postpartum healthcare follow up. The sample size of variable B differs by one patient from variable A. This patient’s healthcare follow up status was to be determined at the time of data collection.

It is important to note that variable A, referral time, is the not the outcome of interest, or dependent variable, while also evaluating if patients met with the reproductive psychiatrist, but rather the predictor, or independent variable. This is due to the temporal relationship of referral preceding meeting with the psychiatrist – thus meeting with the psychiatrist is the outcome of interest in this case. In all other Chi Squared tests between referral time and the independent variables listed in *Variables*, referral time is the outcome of interest, or dependent variable. This is because the independent variables noted in *Variables* are established before the time a patient may have been referred to psychiatry, making each independent variable the predictor or influence of referral time.

## Results

In this study, 62% of patients had met with the reproductive psychiatrist. Of these patients, 50.7% were referred prenatally and 80.6% of these patients had met with the reproductive psychiatrist. In comparison, of the 35.2% of patients who were referred to the reproductive psychiatrist in the postpartum period, only 56% had met with the psychiatrist. Of the patients with unknown referral time, 14.1%, only 10% had met with the psychiatrist.

**Fig. 1:**
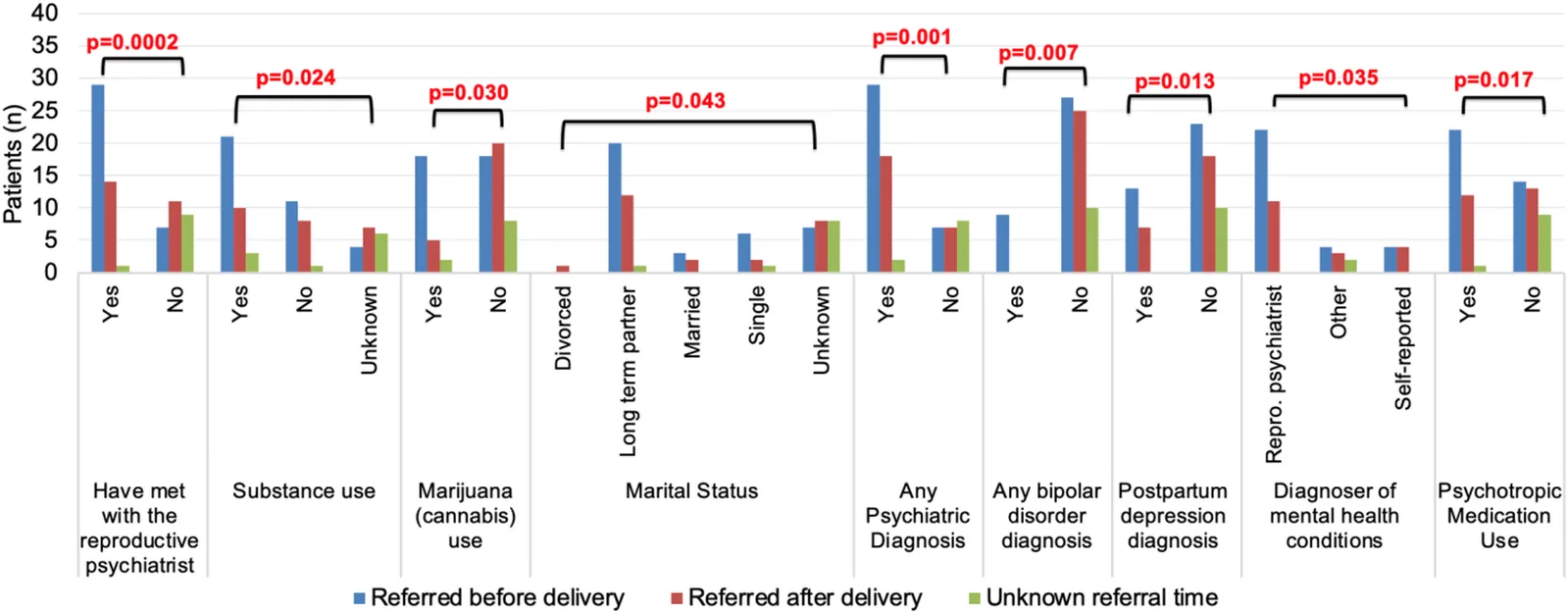
Variables affecting psychiatric referral time in obstetric patients

These results indicate that patients referred prenatally had 3.26 times higher odds of meeting with the reproductive psychiatrist (*N* = 44, 62%) compared to those referred postnatally (*p* = 0.0002). Patients with a history of substance use, marijuana use, presence of long-term partners, use of prescribed psychotropic medication, history of any bipolar disorder, and had their diagnosis made by the reproductive psychiatrist were associated with higher rates of prenatal psychiatry referral, while patients diagnosed with postpartum depression were associated with higher rates of postpartum psychiatry referral as seen in Table and Fig. 1.

**Table 1:** Variables affecting psychiatric referral time in obstetric patients

Variable	Outcome	Referred before delivery	Referred after delivery	Unknown referral time	Chi Squared p value
		%	%	%	
Have met with the reproductive psychiatrist	Yes	80.6	56	10	0.0002
	No	19.4	44	90	
Substance use	Yes	58.3	40	30	0.024
	No	30.6	32	10	
	Unknown	11.1	28	60	
Marijuana (cannabis) use	Yes	50	20	20	0.030
	No	50	80	80	
Marital status	Divorced	0	4	0	0.043
	Long term partner	55.6	48	10	
	Married	8.3	8	0	
	Single	16.7	8	10	
	unknown	19.4	32	80	
Any psychiatric diagnosis	Yes	80.6	72	20	0.001
	No	19.4	28	80	
Any bipolar disorder diagnosis	Yes	25	0	0	0.007
	No	75	100	100	
Postpartum depression diagnosis	Yes	36.1	28	0	0.013
	No	63.9	72	100	
Diagnoser of mental health conditions	Dedicated reproductivepsychiatrist	73.3	61.1	0	0.035
	Other	13.3	16.7	100	
	Self-reported	13.3	22.2	0	
Psychotropic medication use	Yes	61.1	48	10	0.017
	No	38.9	52	90	

Table 1: Demonstrates the relationship between variable A, referral time, and independent variables which are noted in the first column starting at “substance use”, not including “have met with the reproductive psychiatrist”. The percentage of patients who had prenatal psychiatric referral, postpartum psychiatric referral, as well as patients with unknown psychiatric referral time, are listed next to the independent variable being analyzed. To reiterate, as mentioned in, Statistical Analyses, referral time becomes the independent variable or influence when evaluating referral time against “have met with the reproductive psychiatrist”, which is the dependent variable. This is due to the temporal relationship of referral preceding meeting the with the psychiatrist. An example of how to interpret this table is noted below:

Of patients who were referred to psychiatry prenatally, 80.6% have a psychiatric diagnosis whereas 19.4% of patients who were referred prenatally do not have a psychiatric diagnosis. Of patients who were referred to psychiatry postnatally, 72% have a psychiatric diagnosis whereas 28% of patients who were referred postnatally do not have a psychiatric diagnosis. Of patients who had an unknown referral time, 20% have a psychiatric diagnosis whereas 80% of patients with an unknown referral time do not have a psychiatric diagnosis.

Figure 1: Demonstrates the relationship between variable A, referral time, and independent variables which each have a bar graph. As mentioned above, referral time is the independent variable when evaluating it against “have met with the reproductive psychiatrist” due to the temporal relationship of referral preceding meeting the with the psychiatrist. This provides a visual representation of what is displayed in Table 1. Patients are listed here in numbers, n, rather than percentages as they are in Table 1.

A key finding of this study is that patients who met with the reproductive psychiatrist were associated with lower rates of being lost to postpartum healthcare follow up. Overall, 51.4% of our patients were lost to follow up, and of these patients, 47.2% met with the psychiatrist. Of the 48.6% of patients who were not lost to follow up, 76.5% of these patients met with the psychiatrist. Based on these results, the odds of patients who met with the reproductive psychiatrist (*N* = 43, 61.4%) being lost to postpartum healthcare follow up were 3.63 times lower compared to the odds of patients who did not meet with the psychiatrist (*p* = 0.012).

**Fig. 2:**
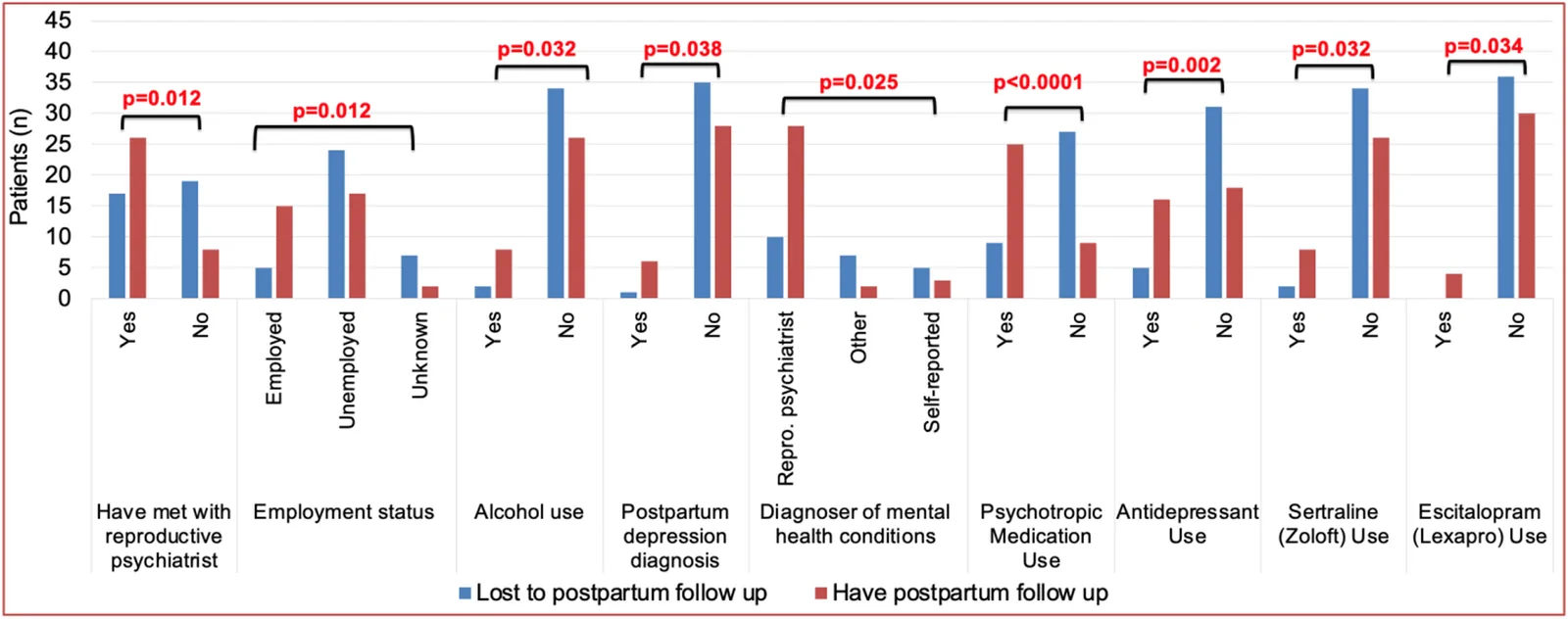
Variables affecting postpartum healthcare follow up

Furthermore, patients who used alcohol, had a diagnosis of postpartum depression, had their mental health conditions diagnosed by the reproductive psychiatrist, were taking a psychotropic medication, particularly antidepressants including sertraline and escitalopram, were associated with lower rates of being lost to postpartum healthcare follow up. Patients who were unemployed were associated with higher rates being lost to postpartum healthcare follow up. This data is seen in Table and Fig. 2.

Table 2: Demonstrates the relationship between variable B, lost to postpartum healthcare follow up, and independent variables which are noted in the first column. The percentage of patients who were lost to postpartum follow up, as well as percentage of patients who had postpartum follow up, are listed next to the independent variable being analyzed. An example of how to interpret this table is listed below: 

Of patients who were lost to postpartum healthcare follow up, 2.8% have a postpartum depression diagnosis whereas 97.2% of patients who were lost to follow up do not have a postpartum depression diagnosis. Of patients who had postpartum healthcare follow up, 17.6% have a postpartum depression diagnosis whereas 82.4% of patients who had postpartum follow up do not have a postpartum depression diagnosis.

**Table 2:** Variables affecting postpartum healthcare follow up

Variable	Outcome	Lost to postpartum follow up	Has postpartum follow up	Chi Squared p value
		%	%	
Have met with the reproductive psychiatrist	Yes	47.2	76.5	0.012
	No	52.8	23.5	
Employment status	Employed	13.9	44.1	0.012
	Unemployed	66.7	50	
	Unknown	19.4	5.9	
Alcohol use	Yes	5.6	23.5	0.032
	No	94.4	76.5	
Postpartum depression diagnosis	Yes	2.8	17.6	0.038
	No	97.2	82.4	
Diagnoser of mental health conditions	Dedicated reproductive psychiatrist	45.5	81.5	0.025
	Other	31.8	7.4	
	Self-reported	22.7	11.1	
Psychotropic medication use	Yes	25	73.5	<0.0001
	No	75	26.5	
Antidepressant use	Yes	13.9	47.1	0.002
	No	86.1	52.9	
Sertraline (Zoloft) use	Yes	5.6	23.5	0.032
	No	94.4	76.5	
Escitalopram (Lexapro) use	Yes	0	11.8	0.034
	No	100	88.2	

Figure 2: Demonstrates the relationship between variable B, lost to postpartum healthcare follow up, and independent variables which each have a bar graph. This provides a visual representation of what is displayed in Table 2. Patients are listed here in numbers, n, rather than percentages as they are in Table 2.

## Discussion

The most notable findings of this study are (1) Psychiatric referral in the prenatal period is associated with higher rates of meeting with the reproductive psychiatrist compared to referral in the postpartum period, and (2) Meeting with the psychiatrist is associated with lower rates of patients who were lost to healthcare follow up after delivery of their child. Other notable findings as they relate to variable A, referral time, and variable B, lost to postpartum healthcare follow up, are below.

### Psychiatric Referral Time

Timing of referral to psychiatric care is crucial in terms of ability to medically intervene via diagnosis and treatment of mental illnesses in obstetric patients. Within this study, prenatal psychiatric referral was associated with higher rates of seeing a reproductive psychiatrist compared to postpartum referral. This pattern may reflect the fact that patients who maintain consistent prenatal care are more closely engaged with the healthcare system, thereby increasing the likelihood that psychiatric needs are identified. Consequently, greater adherence to healthcare recommendations represents a potential confounding factor in this association.

Sociologic factors such as history of substance use, particularly marijuana use, and presence of long-term partners were associated with higher rates of prenatal psychiatric referral. To expand upon the effects of sociologic factors, another study has noted that perinatal substance use increases the likelihood of perinatal depression and anxiety(Pentecost et al., 2021). The linkage between the two conditions may prompt providers to refer patients based on symptoms of mental illness in the setting of substance use, although the relationship between substance use and mental illness was not explicitly elucidated in our study. Thus, concurrent mental illness may be a confounding factor as to why patients with a history of substance use had higher rates of being referred to reproductive psychiatry prenatally. In terms of partnership, a strong social support increases the rate of antenatal appointment attendance(Daniels et al., 2006; Haddrill et al., 2014; Mehta et al., 2017). In this study, it was discovered that patients with long-term partners were associated with higher rates of being referred to psychiatry prenatally compared to those without partners, possibly due to more regular attendance of antenatal appointments.

Patients taking a prescribed psychotropic medication and patients with a history psychiatric diagnosis, particularly bipolar disorder, were associated with higher rates of prenatal psychiatric referral. These situations shed light on what seem to be clear indications for an obstetrician to seek out additional psychiatric care for their patients. In the setting of psychotropic medication use, for bipolar disorder or another mental illness, the benefit of a psychiatrist's input outweighs the uncertainty of whether or not to outsource care. This may be attributed to the need to assess risks and benefits of using psychotropic medications in pregnancy in addition to close monitoring, which an obstetrician may not feel comfortable or confident managing. In the setting of bipolar disorder, all the above is true plus the need to assess which medication(s) may be necessary to keep the patient stable throughout pregnancy. An example of medication management may be choosing lithium as the medication of choice in the setting of a patient who is lithium responsive and at risk for severe episodes or suicidal ideation(Singh & Deep, hiatris^2023^). On the other hand, it should be noted that the association between prenatal psychiatric referral and psychotropic medication use may be due to the fact that patients in our study were prescribed such medications by the reproductive psychiatrist, which may have occurred in the prenatal period.

Postpartum psychiatric referral was more common in patients diagnosed with postpartum depression. This can be explained by the fact that patients may have developed symptoms after giving birth thus necessitating a psychiatric referral. Of patients in this study who were referred to psychiatry after birth, 24% had a diagnosis of postpartum depression. A new diagnosis or suspicion of postpartum depression is a case where it is appropriate to refer to psychiatry after the patient gives birth, although a history of postpartum depression in prior pregnancies should be considered when deciding if to refer to psychiatry prenatally. To elaborate, it has been elucidated that women with a history of postpartum depression have a significantly higher rate of postpartum depression with their second pregnancy compared to patients without a postpartum depression history. More specifically, patients had a 29.6 times higher rate of recurrent postpartum depression after the birth of their second child if they were taking an antidepressant medication after their first birth, and up to 46.4 times higher rate of postpartum depression after birth of their second child if they only had a hospital contact after their first birth, without any pharmacological treatment(Rasmussen et al., 2017). These results alone can validate a prenatal psychiatric referral in patients with a history of PPD.

While it cannot be understated that prenatal referral to reproductive psychiatry may be beneficial for a multitude of reasons as stated above, it is important to emphasize that 62% of patients who were referred to psychiatry met with the psychiatrist regardless of whether they were referred prenatally or postnatally.

### Postpartum Healthcare Follow Up

As stated above, this study found an association in which patients who met with the reproductive psychiatrist had lower rates of being lost to postpartum healthcare follow up compared with those who did not meet with the psychiatrist. It is important to note that this association may be confounded by the fact that patients who attend specialist appointments are, by definition, more engaged with the healthcare system, which in turn reduces the chance of being lost to follow-up.

Furthermore, patients who had their mental health conditions diagnosed by the psychiatrist and who were taking a selective serotonin reuptake inhibitor (SSRI), particularly sertraline or escitalopram, were associated with lower rates of being lost to follow up, while patients not taking psychotropic medications were associated with higher rates of being lost to follow up. The respective difference of SSRI use versus no psychotropic medication use and its effect on follow up may be confounded by the fact that a portion of patients that were prescribed SSRIs had received the prescription from the psychiatrist in our study, although this is not the case for all patients in this study. On the other hand, SSRIs have been shown to not only treat postpartum depression(Frieder et al., 2019), but also decrease long term maternal mental health problems in patients with postpartum depression(Liu et al., 2023), which may explain the necessity of patients to follow up for prescription refills. While considering SSRI use and follow up, it must also be noted that patients in our study were not exclusively prescribed SSRIs for postpartum depression, but also for mental illness during pregnancy. This creates an interesting dynamic between the use of SSRIs during pregnancy and postpartum healthcare follow up when considering the lack of randomized control trials of SSRI efficacy during pregnancy. The association of lower rates of being lost to postpartum healthcare follow up as it relates to SSRI use both during and after pregnancy shown by our study demonstrates the possible efficacy of SSRIs during pregnancy and the medications’ impact on postpartum care, although further research is needed to expand upon this.

Patients diagnosed with postpartum depression were associated with lower rates of being lost to follow up. It is likely that most patients in our study would have had to attend a postpartum visit to receive a diagnosis of postpartum depression, which explains this finding.

We identified that certain sociologic factors, like alcohol use and employment, may influence rates of being lost to postpartum healthcare follow up. Unemployment is associated with higher rates of being lost to follow up, which has been somewhat established in prior studies that have demonstrated low income patients have lower postpartum attendance rates(Howell et al., 2020; Tenfelde et al., 2023). Unlike employment, alcohol use had the opposite and, to some extent, unexpected association with follow up. In our study, patients with alcohol use were associated with lower rates of being lost to follow up. While our study did not find a statistically significant relationship between referral time and alcohol use, patients with alcohol use may have other confounding, concurrent factors that would increase their chances of a prenatal psychiatric referral such as marijuana use, history of psychotropic medication use, having a long-term partner, etc. The aforementioned factors were expanded on above when discussing referral time variables. Similarly, this finding may be explained by another result of our study which is that patients who used substances were associated with higher rates of being referred to psychiatry before delivery. This explanation would be viable if the substances included alcohol.

### Future Recommendations

The variability of standardized psychiatric screening in the prenatal period is an issue that unquestionably contributes to a lack of awareness of the need for psychiatric intervention in many obstetric patients. This variability arises when healthcare providers follow inconsistent screening schedules and practices, and when patients miss or do not regularly attend prenatal care appointments. Screening ultimately impacts which obstetric patients are referred to psychiatry and when they are referred. By assessing inhibitors of screening on a practice level, changes can be implemented to mitigate such factors, which may allow for greater rates of necessary psychiatric referral. Inhibitors to screening that can be evaluated include lack of personnel to screen patients, time constraints, limited resources to refer to if there is a positive screen, provider indifference to screening, etc. On a guideline level, standardization of screening at fixed time points may lead to increased cognizance of psychiatric needs in obstetric patients.

On a similar note, inability to access screening results may leave providers without the knowledge that a patient may require psychiatric intervention. There is not a dedicated place to input an Edinburgh Postnatal Depression (EPDS) score in the electronic medical record used at TUHS, EPIC, which leaves the provider without quick access to the data. Adding either a dedicated space for the score in EPIC or within a note template may bring awareness to the EPDS score via accessibility and reduce the burden of searching for a physical paper or scanned document in EPIC.

A benefit of this study may be to bring awareness and create an opportunity to educate providers about which individual factors may influence their willingness to refer obstetric patients to psychiatry. By understanding such factors, providers can internally reflect to identify what factors seem clearer as to when to refer to psychiatry, and on the other hand, which factors are more obscure and may cause more uncertainty. Synthesis of the results of this study and education by a psychiatrist may be beneficial to increase both confidence of providers and likelihood of psychiatric referral in obstetric patients.

### Limitations

While this study is a strong starting point for understanding the relationship between psychiatric referral time/postpartum healthcare follow up and psychiatric intervention, a study with a larger sample size could provide even further evidence of the substantial positive influence of early psychiatric intervention in the care of obstetric patients as it relates to postpartum healthcare follow up. Additionally, the ability to account for clear duality of referral times, before delivery or after delivery, may have influenced the results of this study. There were 10 patients (14.1%) who had an unclear referral time, which may possibly account for the absence of a direct association between prenatal psychiatric referral and having postpartum healthcare follow up.

While collecting data, it was not specified whether “follow up” was specific to obstetrics, psychiatry, and/ or primary care, which may give further insight into how entrance into psychiatric care influences postpartum healthcare follow up. Similarly, the timing of initiation of psychotropic medication for patients was not specified during data collection. Data about if and when patients were screened for psychiatric illness at prenatal appointments would have been useful in further understanding how screening relates to psychiatric referral time and eventual postpartum healthcare follow up in this set of patients.

Furthermore, this study evaluated a multitude of medical, sociologic and cultural variables against two main variables, referral time and if patients were lost to postpartum healthcare follow up, without accounting for potential confounding effects by comparing the independent variables of each test against each other. A different statistically analysis, multinomial logistic regression model, could have been used to evaluate the independent variables together. In setting of the numerous independent variables, this option seemed to be beyond the scope of this study. To offset the lack of evaluation of interdependence between independent variables, confounding factors were addressed in the discussion.

It cannot be understated that patients who are connected to healthcare at the outset of pregnancy are more likely to attend appointments, which leaves out a large portion of the population who do not have the same connectedness to healthcare.

## Conclusion

In conclusion, there are identifiable factors that influence psychiatric referral time and postpartum healthcare follow up in obstetric patients. This study gives further insight into how timing of psychiatric referral in obstetric patients can impact attendance rates of these appointments, and how successful psychiatric intervention can potentially lead to increase connectedness to healthcare in the postpartum period. Knowledge of these findings may encourage providers to consider psychiatric referral more readily. Similarly, by understanding individual factors that independently influence psychiatric referral time and healthcare follow up rates, obstetric providers can bring awareness to what impacts their decision, or lack thereof, to refer patients to psychiatry.

## Supplementary Information

Below is the link to the electronic supplementary material.

Supplementary Material 1 (179 KB)

## Data Availability

Data is provided in the supplementary information files.
