# Supplementary material for: Assessing Factors that Influence Psychiatric Referral Time and Postpartum Follow-Up in Obstetric Patients
Source: Community Ment Health J. 2026 Apr 30;62(7):1416–25. doi: 10.1007/s10597-026-01608-7 (PMC13585855; doi:10.1007/s10597-026-01608-7)
Supplement: Supplementary file 1 [file 10597_2026_1608_MOESM1_ESM.docx]

|  | | | **Lost to follow up?** | |  |
| --- | --- | --- | --- | --- | --- |
| **Variable** | **NMiss** | **Overall (n=70)** | **Yes (n=36)** | **No (n=34)** | **Chisq or T-test p-value** |
| Have seen Dr. Tabi, N (%) | 0 |  |  |  | **0.012** |
| No |  | 27 (38.6%) | 19 (52.8%) | 8 (23.5%) |  |
| Yes |  | 43 (61.4%) | 17 (47.2%) | 26 (76.5%) |  |
| When Referred?, N (%) | 10 |  |  |  | 0.79 |
| After birth |  | 25 (41.7%) | 13 (43.3%) | 12 (40.0%) |  |
| Before birth |  | 35 (58.3%) | 17 (56.7%) | 18 (60.0%) |  |
| Prenatal EPDS SI, N (%) | 22 |  |  |  | 0.65 |
| Hardly ever |  | 9 (18.8%) | 3 (14.3%) | 6 (22.2%) |  |
| Never/Denies |  | 35 (72.9%) | 17 (81.0%) | 18 (66.7%) |  |
| Sometimes |  | 3 (6.3%) | 1 (4.8%) | 2 (7.4%) |  |
| Yes, quite often |  | 1 (2.1%) | 0 (0.0%) | 1 (3.7%) |  |
| Postpartum EPDS SI, N (%) | 39 |  |  |  |  |
| Never/Denies |  | 31 (100.0%) | 1 (100.0%) | 30 (100.0%) |  |
| Age, N | 0 | 70 | 36 | 34 | **0.073** |
| Mean (SD) |  | 27.8 (6.4) | 26.4 (5.7) | 29.2 (7.0) |  |
| Median (IQR) |  | 27.5 (22.0, 33.0) | 25.5 (21.5, 32.0) | 30.5 (24.0, 35.0) |  |
| Median (Range) |  | 27.5 (17.0, 39.0) | 25.5 (18.0, 38.0) | 30.5 (17.0, 39.0) |  |
| Ethnicity (choice=Hispanic/Latino of any race), N (%) | 0 |  |  |  | 0.35 |
| Checked |  | 23 (32.9%) | 10 (27.8%) | 13 (38.2%) |  |
| Unchecked |  | 47 (67.1%) | 26 (72.2%) | 21 (61.8%) |  |
| Ethnicity (choice=American Indian or Alaska Native), N (%) | 0 |  |  |  |  |
| Unchecked |  | 70 (100.0%) | 36 (100.0%) | 34 (100.0%) |  |
| Ethnicity (choice=Asian), N (%) | 0 |  |  |  |  |
| Unchecked |  | 70 (100.0%) | 36 (100.0%) | 34 (100.0%) |  |
| Ethnicity (choice=Black or African American), N (%) | 0 |  |  |  | 0.45 |
| Checked |  | 30 (42.9%) | 17 (47.2%) | 13 (38.2%) |  |
| Unchecked |  | 40 (57.1%) | 19 (52.8%) | 21 (61.8%) |  |
| Ethnicity (choice=Native Hawaiian or Other Pacific Islander), N (%) | 0 |  |  |  |  |
| Unchecked |  | 70 (100.0%) | 36 (100.0%) | 34 (100.0%) |  |
| Ethnicity (choice=White), N (%) | 0 |  |  |  | 0.87 |
| Checked |  | 15 (21.4%) | 8 (22.2%) | 7 (20.6%) |  |
| Unchecked |  | 55 (78.6%) | 28 (77.8%) | 27 (79.4%) |  |
| Ethnicity (choice=Unknown), N (%) | 0 |  |  |  | 0.97 |
| Checked |  | 2 (2.9%) | 1 (2.8%) | 1 (2.9%) |  |
| Unchecked |  | 68 (97.1%) | 35 (97.2%) | 33 (97.1%) |  |
| Employed?, N (%) | 0 |  |  |  | **0.012** |
| No |  | 41 (58.6%) | 24 (66.7%) | 17 (50.0%) |  |
| Unknown |  | 9 (12.9%) | 7 (19.4%) | 2 (5.9%) |  |
| Yes |  | 20 (28.6%) | 5 (13.9%) | 15 (44.1%) |  |
| Unhoused?, N (%) | 0 |  |  |  | 0.18 |
| No |  | 65 (92.9%) | 32 (88.9%) | 33 (97.1%) |  |
| Unknown |  | 5 (7.1%) | 4 (11.1%) | 1 (2.9%) |  |
| Substance use?, N (%) | 0 |  |  |  | 0.28 |
| No |  | 20 (28.6%) | 9 (25.0%) | 11 (32.4%) |  |
| Unknown |  | 16 (22.9%) | 11 (30.6%) | 5 (14.7%) |  |
| Yes |  | 34 (48.6%) | 16 (44.4%) | 18 (52.9%) |  |
| Substance use during pregnancy?, N (%) | 36 |  |  |  | 0.80 |
| No |  | 12 (35.3%) | 6 (37.5%) | 6 (33.3%) |  |
| Yes |  | 22 (64.7%) | 10 (62.5%) | 12 (66.7%) |  |
| Substance Type? (choice=Alcohol), N (%) | 0 |  |  |  | **0.032** |
| Checked |  | 10 (14.3%) | 2 (5.6%) | 8 (23.5%) |  |
| Unchecked |  | 60 (85.7%) | 34 (94.4%) | 26 (76.5%) |  |
| Substance Type? (choice=Caffeine), N (%) | 0 |  |  |  | 0.33 |
| Checked |  | 1 (1.4%) | 1 (2.8%) | 0 (0.0%) |  |
| Unchecked |  | 69 (98.6%) | 35 (97.2%) | 34 (100.0%) |  |
| Substance Type? (choice=Cannabis (Marijuana)), N (%) | 0 |  |  |  | 0.67 |
| Checked |  | 25 (35.7%) | 12 (33.3%) | 13 (38.2%) |  |
| Unchecked |  | 45 (64.3%) | 24 (66.7%) | 21 (61.8%) |  |
| Substance Type? (choice=LSD (Lysergic Acid Diethylamide)), N (%) | 0 |  |  |  |  |
| Unchecked |  | 70 (100.0%) | 36 (100.0%) | 34 (100.0%) |  |
| Substance Type? (choice=Psilocybin (Magic Mushrooms)), N (%) | 0 |  |  |  |  |
| Unchecked |  | 70 (100.0%) | 36 (100.0%) | 34 (100.0%) |  |
| Substance Type? (choice=Phencyclidine (PCP)), N (%) | 0 |  |  |  | 0.33 |
| Checked |  | 1 (1.4%) | 1 (2.8%) | 0 (0.0%) |  |
| Unchecked |  | 69 (98.6%) | 35 (97.2%) | 34 (100.0%) |  |
| Substance Type? (choice=Ketamine), N (%) | 0 |  |  |  |  |
| Unchecked |  | 70 (100.0%) | 36 (100.0%) | 34 (100.0%) |  |
| Substance Type? (choice=Inhalants (e.g., paint thinner, gasoline fumes)), N (%) | 0 |  |  |  |  |
| Unchecked |  | 70 (100.0%) | 36 (100.0%) | 34 (100.0%) |  |
| Substance Type? (choice=Opioids (e.g., heroin, prescription pain medications)), N (%) | 0 |  |  |  | 0.94 |
| Checked |  | 6 (8.6%) | 3 (8.3%) | 3 (8.8%) |  |
| Unchecked |  | 64 (91.4%) | 33 (91.7%) | 31 (91.2%) |  |
| Substance Type? (choice=Benzodiazepines (e.g., Xanax, Valium)), N (%) | 0 |  |  |  | 0.95 |
| Checked |  | 4 (5.7%) | 2 (5.6%) | 2 (5.9%) |  |
| Unchecked |  | 66 (94.3%) | 34 (94.4%) | 32 (94.1%) |  |
| Substance Type? (choice=Barbiturates (e.g., Phenobarbital)), N (%) | 0 |  |  |  |  |
| Unchecked |  | 70 (100.0%) | 36 (100.0%) | 34 (100.0%) |  |
| Substance Type? (choice=Non-Benzodiazepine Hypnotics (e.g., Ambien, Lunesta)), N (%) | 0 |  |  |  |  |
| Unchecked |  | 70 (100.0%) | 36 (100.0%) | 34 (100.0%) |  |
| Substance Type? (choice=Amphetamines (e.g., Adderall, methamphetamine)), N (%) | 0 |  |  |  | 0.97 |
| Checked |  | 2 (2.9%) | 1 (2.8%) | 1 (2.9%) |  |
| Unchecked |  | 68 (97.1%) | 35 (97.2%) | 33 (97.1%) |  |
| Substance Type? (choice=Cocaine), N (%) | 0 |  |  |  | 0.94 |
| Checked |  | 6 (8.6%) | 3 (8.3%) | 3 (8.8%) |  |
| Unchecked |  | 64 (91.4%) | 33 (91.7%) | 31 (91.2%) |  |
| Substance Type? (choice=Tobacco (Nicotine)), N (%) | 0 |  |  |  | **0.060** |
| Checked |  | 9 (12.9%) | 2 (5.6%) | 7 (20.6%) |  |
| Unchecked |  | 61 (87.1%) | 34 (94.4%) | 27 (79.4%) |  |
| Substance Type? (choice=Synthetic Cannabinoids (e.g., Spice, K2)), N (%) | 0 |  |  |  |  |
| Unchecked |  | 70 (100.0%) | 36 (100.0%) | 34 (100.0%) |  |
| Substance Type? (choice=Synthetic Cathinones (e.g., Bath Salts)), N (%) | 0 |  |  |  |  |
| Unchecked |  | 70 (100.0%) | 36 (100.0%) | 34 (100.0%) |  |
| Substance Type? (choice=Other or Unknown Substances), N (%) | 0 |  |  |  |  |
| Unchecked |  | 70 (100.0%) | 36 (100.0%) | 34 (100.0%) |  |
| Method of Substance Use? (choice=Inhalation (e.g., smoking, vaping)), N (%) | 0 |  |  |  | 0.30 |
| Checked |  | 1 (1.4%) | 0 (0.0%) | 1 (2.9%) |  |
| Unchecked |  | 69 (98.6%) | 36 (100.0%) | 33 (97.1%) |  |
| Method of Substance Use? (choice=Oral Ingestion (e.g., swallowing pills or liquids)), N (%) | 0 |  |  |  |  |
| Unchecked |  | 70 (100.0%) | 36 (100.0%) | 34 (100.0%) |  |
| Method of Substance Use? (choice=Intranasal (e.g., snorting or sniffing)), N (%) | 0 |  |  |  |  |
| Unchecked |  | 70 (100.0%) | 36 (100.0%) | 34 (100.0%) |  |
| Method of Substance Use? (choice=Intravenous (IV) Injection), N (%) | 0 |  |  |  |  |
| Unchecked |  | 70 (100.0%) | 36 (100.0%) | 34 (100.0%) |  |
| Method of Substance Use? (choice=Intramuscular (IM) Injection), N (%) | 0 |  |  |  |  |
| Unchecked |  | 70 (100.0%) | 36 (100.0%) | 34 (100.0%) |  |
| Method of Substance Use? (choice=Subcutaneous (SC) Injection), N (%) | 0 |  |  |  |  |
| Unchecked |  | 70 (100.0%) | 36 (100.0%) | 34 (100.0%) |  |
| Method of Substance Use? (choice=Transdermal (e.g., patches)), N (%) | 0 |  |  |  |  |
| Unchecked |  | 70 (100.0%) | 36 (100.0%) | 34 (100.0%) |  |
| Method of Substance Use? (choice=Rectal (e.g., suppositories)), N (%) | 0 |  |  |  |  |
| Unchecked |  | 70 (100.0%) | 36 (100.0%) | 34 (100.0%) |  |
| Method of Substance Use? (choice=Sublingual (under the tongue)), N (%) | 0 |  |  |  |  |
| Unchecked |  | 70 (100.0%) | 36 (100.0%) | 34 (100.0%) |  |
| Method of Substance Use? (choice=Transbuccal (between the cheek and gum)), N (%) | 0 |  |  |  |  |
| Unchecked |  | 70 (100.0%) | 36 (100.0%) | 34 (100.0%) |  |
| History of abuse?, N (%) | 0 |  |  |  | 0.17 |
| No |  | 17 (24.3%) | 6 (16.7%) | 11 (32.4%) |  |
| Unknown |  | 30 (42.9%) | 19 (52.8%) | 11 (32.4%) |  |
| Yes |  | 23 (32.9%) | 11 (30.6%) | 12 (35.3%) |  |
| Relationship Status, N (%) | 0 |  |  |  | 0.46 |
| Divorced |  | 1 (1.4%) | 1 (2.8%) | 0 (0.0%) |  |
| Has long-term partner |  | 33 (47.1%) | 14 (38.9%) | 19 (55.9%) |  |
| Married |  | 5 (7.1%) | 2 (5.6%) | 3 (8.8%) |  |
| Single |  | 9 (12.9%) | 5 (13.9%) | 4 (11.8%) |  |
| Unknown |  | 22 (31.4%) | 14 (38.9%) | 8 (23.5%) |  |
| Education Level, N (%) | 0 |  |  |  | 0.23 |
| Associates degree |  | 2 (2.9%) | 0 (0.0%) | 2 (5.9%) |  |
| Bachelors degree |  | 1 (1.4%) | 0 (0.0%) | 1 (2.9%) |  |
| Below High School |  | 10 (14.3%) | 6 (16.7%) | 4 (11.8%) |  |
| Graduate Degree |  | 2 (2.9%) | 0 (0.0%) | 2 (5.9%) |  |
| High School |  | 16 (22.9%) | 7 (19.4%) | 9 (26.5%) |  |
| Unknown |  | 39 (55.7%) | 23 (63.9%) | 16 (47.1%) |  |
| Previous IP hospitalizations?, N (%) | 1 |  |  |  | 0.87 |
| Found |  | 12 (17.4%) | 6 (16.7%) | 6 (18.2%) |  |
| Not Found |  | 57 (82.6%) | 30 (83.3%) | 27 (81.8%) |  |
| Psychiatric Diagnosis (choice=None), N (%) | 0 |  |  |  | **0.058** |
| Checked |  | 22 (31.4%) | 15 (41.7%) | 7 (20.6%) |  |
| Unchecked |  | 48 (68.6%) | 21 (58.3%) | 27 (79.4%) |  |
| Psychiatric Diagnosis (choice=Bipolar Disorders), N (%) | 0 |  |  |  | **0.060** |
| Checked |  | 9 (12.9%) | 2 (5.6%) | 7 (20.6%) |  |
| Unchecked |  | 61 (87.1%) | 34 (94.4%) | 27 (79.4%) |  |
| Psychiatric Diagnosis (choice=Major Depressive Disorder (MDD)), N (%) | 0 |  |  |  | 0.58 |
| Checked |  | 27 (38.6%) | 15 (41.7%) | 12 (35.3%) |  |
| Unchecked |  | 43 (61.4%) | 21 (58.3%) | 22 (64.7%) |  |
| Psychiatric Diagnosis (choice=Schizophrenia and Other Psychotic Disorders), N (%) | 0 |  |  |  | 0.69 |
| Checked |  | 5 (7.1%) | 3 (8.3%) | 2 (5.9%) |  |
| Unchecked |  | 65 (92.9%) | 33 (91.7%) | 32 (94.1%) |  |
| Psychiatric Diagnosis (choice=Generalized Anxiety Disorder (GAD)), N (%) | 0 |  |  |  | 0.50 |
| Checked |  | 24 (34.3%) | 11 (30.6%) | 13 (38.2%) |  |
| Unchecked |  | 46 (65.7%) | 25 (69.4%) | 21 (61.8%) |  |
| Psychiatric Diagnosis (choice=Borderline Personality Disorder), N (%) | 0 |  |  |  | 0.33 |
| Checked |  | 1 (1.4%) | 1 (2.8%) | 0 (0.0%) |  |
| Unchecked |  | 69 (98.6%) | 35 (97.2%) | 34 (100.0%) |  |
| Psychiatric Diagnosis (choice=Substance Use Disorder), N (%) | 0 |  |  |  | 0.51 |
| Checked |  | 8 (11.4%) | 5 (13.9%) | 3 (8.8%) |  |
| Unchecked |  | 62 (88.6%) | 31 (86.1%) | 31 (91.2%) |  |
| Psychiatric Diagnosis (choice=Obsessive Compulsive Disorder (OCD)), N (%) | 0 |  |  |  |  |
| Unchecked |  | 70 (100.0%) | 36 (100.0%) | 34 (100.0%) |  |
| Psychiatric Diagnosis (choice=Posttraumatic Stress Disorder (PTSD)), N (%) | 0 |  |  |  | 0.50 |
| Checked |  | 20 (28.6%) | 9 (25.0%) | 11 (32.4%) |  |
| Unchecked |  | 50 (71.4%) | 27 (75.0%) | 23 (67.6%) |  |
| Psychiatric Diagnosis (choice=postpartum depressive disorder), N (%) | 0 |  |  |  | **0.038** |
| Checked |  | 7 (10.0%) | 1 (2.8%) | 6 (17.6%) |  |
| Unchecked |  | 63 (90.0%) | 35 (97.2%) | 28 (82.4%) |  |
| Diagnoser, N (%) | 21 |  |  |  | **0.025** |
| Dr. Tabi |  | 32 (65.3%) | 10 (45.5%) | 22 (81.5%) |  |
| Other |  | 9 (18.4%) | 7 (31.8%) | 2 (7.4%) |  |
| Self-reported |  | 8 (16.3%) | 5 (22.7%) | 3 (11.1%) |  |
| Bipolar Disorders (choice=Bipolar I Disorder), N (%) | 0 |  |  |  | 0.97 |
| Checked |  | 2 (2.9%) | 1 (2.8%) | 1 (2.9%) |  |
| Unchecked |  | 68 (97.1%) | 35 (97.2%) | 33 (97.1%) |  |
| Bipolar Disorders (choice=Bipolar II Disorder), N (%) | 0 |  |  |  | 0.30 |
| Checked |  | 1 (1.4%) | 0 (0.0%) | 1 (2.9%) |  |
| Unchecked |  | 69 (98.6%) | 36 (100.0%) | 33 (97.1%) |  |
| Bipolar Disorders (choice=Cyclothymic Disorder (Cyclothymia)), N (%) | 0 |  |  |  |  |
| Unchecked |  | 70 (100.0%) | 36 (100.0%) | 34 (100.0%) |  |
| Bipolar Disorders (choice=Other Specified Bipolar and Related Disorder), N (%) | 0 |  |  |  | 0.30 |
| Checked |  | 1 (1.4%) | 0 (0.0%) | 1 (2.9%) |  |
| Unchecked |  | 69 (98.6%) | 36 (100.0%) | 33 (97.1%) |  |
| Bipolar Disorders (choice=Unspecified Bipolar and Related Disorder), N (%) | 0 |  |  |  | 0.14 |
| Checked |  | 2 (2.9%) | 0 (0.0%) | 2 (5.9%) |  |
| Unchecked |  | 68 (97.1%) | 36 (100.0%) | 32 (94.1%) |  |
| Substance Use Disorders (per DSM5) (choice=Alcohol Use Disorder), N (%) | 0 |  |  |  | 0.33 |
| Checked |  | 1 (1.4%) | 1 (2.8%) | 0 (0.0%) |  |
| Unchecked |  | 69 (98.6%) | 35 (97.2%) | 34 (100.0%) |  |
| Substance Use Disorders (per DSM5) (choice=Caffeine Use Disorder), N (%) | 0 |  |  |  |  |
| Unchecked |  | 70 (100.0%) | 36 (100.0%) | 34 (100.0%) |  |
| Substance Use Disorders (per DSM5) (choice=Cannabis Use Disorder), N (%) | 0 |  |  |  | **0.085** |
| Checked |  | 3 (4.3%) | 3 (8.3%) | 0 (0.0%) |  |
| Unchecked |  | 67 (95.7%) | 33 (91.7%) | 34 (100.0%) |  |
| Substance Use Disorders (per DSM5) (choice=Hallucinogen Use Disorder), N (%) | 0 |  |  |  | 0.33 |
| Checked |  | 1 (1.4%) | 1 (2.8%) | 0 (0.0%) |  |
| Unchecked |  | 69 (98.6%) | 35 (97.2%) | 34 (100.0%) |  |
| Substance Use Disorders (per DSM5) (choice=Inhalant Use Disorder), N (%) | 0 |  |  |  |  |
| Unchecked |  | 70 (100.0%) | 36 (100.0%) | 34 (100.0%) |  |
| Substance Use Disorders (per DSM5) (choice=Opioid Use Disorder), N (%) | 0 |  |  |  | 0.97 |
| Checked |  | 2 (2.9%) | 1 (2.8%) | 1 (2.9%) |  |
| Unchecked |  | 68 (97.1%) | 35 (97.2%) | 33 (97.1%) |  |
| Substance Use Disorders (per DSM5) (choice=Sedative, Hypnotic, or Anxiolytic Use Disorder), N (%) | 0 |  |  |  | 0.33 |
| Checked |  | 1 (1.4%) | 1 (2.8%) | 0 (0.0%) |  |
| Unchecked |  | 69 (98.6%) | 35 (97.2%) | 34 (100.0%) |  |
| Substance Use Disorders (per DSM5) (choice=Stimulant Use Disorder), N (%) | 0 |  |  |  | 0.14 |
| Checked |  | 2 (2.9%) | 0 (0.0%) | 2 (5.9%) |  |
| Unchecked |  | 68 (97.1%) | 36 (100.0%) | 32 (94.1%) |  |
| Substance Use Disorders (per DSM5) (choice=Tobacco Use Disorder), N (%) | 0 |  |  |  | 0.52 |
| Checked |  | 3 (4.3%) | 1 (2.8%) | 2 (5.9%) |  |
| Unchecked |  | 67 (95.7%) | 35 (97.2%) | 32 (94.1%) |  |
| Substance Use Disorders (per DSM5) (choice=Other (or Unknown) Substance Use Disorder), N (%) | 0 |  |  |  |  |
| Unchecked |  | 70 (100.0%) | 36 (100.0%) | 34 (100.0%) |  |
| Substance Use Disorders (per DSM5) (choice=Alcohol Intoxication), N (%) | 0 |  |  |  |  |
| Unchecked |  | 70 (100.0%) | 36 (100.0%) | 34 (100.0%) |  |
| Substance Use Disorders (per DSM5) (choice=Caffeine Intoxication), N (%) | 0 |  |  |  |  |
| Unchecked |  | 70 (100.0%) | 36 (100.0%) | 34 (100.0%) |  |
| Substance Use Disorders (per DSM5) (choice=Cannabis Intoxication), N (%) | 0 |  |  |  |  |
| Unchecked |  | 70 (100.0%) | 36 (100.0%) | 34 (100.0%) |  |
| Substance Use Disorders (per DSM5) (choice=Stimulant Intoxication (e.g., amphetamines, cocaine)), N (%) | 0 |  |  |  |  |
| Unchecked |  | 70 (100.0%) | 36 (100.0%) | 34 (100.0%) |  |
| Substance Use Disorders (per DSM5) (choice=Opioid Intoxication), N (%) | 0 |  |  |  |  |
| Unchecked |  | 70 (100.0%) | 36 (100.0%) | 34 (100.0%) |  |
| Substance Use Disorders (per DSM5) (choice=Sedative, Hypnotic, or Anxiolytic Intoxication), N (%) | 0 |  |  |  |  |
| Unchecked |  | 70 (100.0%) | 36 (100.0%) | 34 (100.0%) |  |
| Substance Use Disorders (per DSM5) (choice=Hallucinogen Intoxication), N (%) | 0 |  |  |  |  |
| Unchecked |  | 70 (100.0%) | 36 (100.0%) | 34 (100.0%) |  |
| Substance Use Disorders (per DSM5) (choice=Inhalant Intoxication), N (%) | 0 |  |  |  |  |
| Unchecked |  | 70 (100.0%) | 36 (100.0%) | 34 (100.0%) |  |
| Substance Use Disorders (per DSM5) (choice=Caffeine Intoxication), N (%) | 0 |  |  |  |  |
| Unchecked |  | 70 (100.0%) | 36 (100.0%) | 34 (100.0%) |  |
| Substance Use Disorders (per DSM5) (choice=Other (or Unknown) Substance Intoxication), N (%) | 0 |  |  |  |  |
| Unchecked |  | 70 (100.0%) | 36 (100.0%) | 34 (100.0%) |  |
| Substance Use Disorders (per DSM5) (choice=Alcohol Withdrawal), N (%) | 0 |  |  |  |  |
| Unchecked |  | 70 (100.0%) | 36 (100.0%) | 34 (100.0%) |  |
| Substance Use Disorders (per DSM5) (choice=Caffeine Withdrawal), N (%) | 0 |  |  |  |  |
| Unchecked |  | 70 (100.0%) | 36 (100.0%) | 34 (100.0%) |  |
| Substance Use Disorders (per DSM5) (choice=Cannabis Withdrawal), N (%) | 0 |  |  |  |  |
| Unchecked |  | 70 (100.0%) | 36 (100.0%) | 34 (100.0%) |  |
| Substance Use Disorders (per DSM5) (choice=Stimulant Withdrawal (e.g., amphetamines, cocaine)), N (%) | 0 |  |  |  |  |
| Unchecked |  | 70 (100.0%) | 36 (100.0%) | 34 (100.0%) |  |
| Substance Use Disorders (per DSM5) (choice=Opioid Withdrawal), N (%) | 0 |  |  |  |  |
| Unchecked |  | 70 (100.0%) | 36 (100.0%) | 34 (100.0%) |  |
| Substance Use Disorders (per DSM5) (choice=Sedative, Hypnotic, or Anxiolytic Withdrawal), N (%) | 0 |  |  |  |  |
| Unchecked |  | 70 (100.0%) | 36 (100.0%) | 34 (100.0%) |  |
| Substance Use Disorders (per DSM5) (choice=Hallucinogen Persisting Perception Disorder (not strictly withdrawal but related)), N (%) | 0 |  |  |  |  |
| Unchecked |  | 70 (100.0%) | 36 (100.0%) | 34 (100.0%) |  |
| Substance Use Disorders (per DSM5) (choice=Inhalant Withdrawal), N (%) | 0 |  |  |  |  |
| Unchecked |  | 70 (100.0%) | 36 (100.0%) | 34 (100.0%) |  |
| Substance Use Disorders (per DSM5) (choice=Caffeine Withdrawal), N (%) | 0 |  |  |  |  |
| Unchecked |  | 70 (100.0%) | 36 (100.0%) | 34 (100.0%) |  |
| Substance Use Disorders (per DSM5) (choice=Other (or Unknown) Substance Withdrawal), N (%) | 0 |  |  |  |  |
| Unchecked |  | 70 (100.0%) | 36 (100.0%) | 34 (100.0%) |  |
| Psychotropic Medication (choice=None), N (%) | 0 |  |  |  | **<.0001** |
| Checked |  | 36 (51.4%) | 27 (75.0%) | 9 (26.5%) |  |
| Unchecked |  | 34 (48.6%) | 9 (25.0%) | 25 (73.5%) |  |
| Psychotropic Medication (choice=Antidepressant (e.g., SSRI, SNRI, TCA, MAOIs, etc)), N (%) | 0 |  |  |  | **0.002** |
| Checked |  | 21 (30.0%) | 5 (13.9%) | 16 (47.1%) |  |
| Unchecked |  | 49 (70.0%) | 31 (86.1%) | 18 (52.9%) |  |
| Psychotropic Medication (choice=Antipsychotic (e.g., FGA, SGA)), N (%) | 0 |  |  |  | 0.30 |
| Checked |  | 13 (18.6%) | 5 (13.9%) | 8 (23.5%) |  |
| Unchecked |  | 57 (81.4%) | 31 (86.1%) | 26 (76.5%) |  |
| Psychotropic Medication (choice=Mood Stabilizer (e.g., Lithium, AEDs)), N (%) | 0 |  |  |  |  |
| Unchecked |  | 70 (100.0%) | 36 (100.0%) | 34 (100.0%) |  |
| Psychotropic Medication (choice=Anxiolytic (e.g., Benzos, Atarax, Buspirone)), N (%) | 0 |  |  |  | 0.14 |
| Checked |  | 2 (2.9%) | 0 (0.0%) | 2 (5.9%) |  |
| Unchecked |  | 68 (97.1%) | 36 (100.0%) | 32 (94.1%) |  |
| Psychotropic Medication (choice=Sedative (e.g., Ambien)), N (%) | 0 |  |  |  | 0.30 |
| Checked |  | 1 (1.4%) | 0 (0.0%) | 1 (2.9%) |  |
| Unchecked |  | 69 (98.6%) | 36 (100.0%) | 33 (97.1%) |  |
| Psychotropic Medication (choice=Stimulant), N (%) | 0 |  |  |  | 0.97 |
| Checked |  | 2 (2.9%) | 1 (2.8%) | 1 (2.9%) |  |
| Unchecked |  | 68 (97.1%) | 35 (97.2%) | 33 (97.1%) |  |
| Antidepressants (choice=Fluoxetine (Prozac)), N (%) | 0 |  |  |  | 0.59 |
| Checked |  | 3 (4.3%) | 2 (5.6%) | 1 (2.9%) |  |
| Unchecked |  | 67 (95.7%) | 34 (94.4%) | 33 (97.1%) |  |
| Antidepressants (choice=Sertraline (Zoloft)), N (%) | 0 |  |  |  | **0.032** |
| Checked |  | 10 (14.3%) | 2 (5.6%) | 8 (23.5%) |  |
| Unchecked |  | 60 (85.7%) | 34 (94.4%) | 26 (76.5%) |  |
| Antidepressants (choice=Paroxetine (Paxil)), N (%) | 0 |  |  |  |  |
| Unchecked |  | 70 (100.0%) | 36 (100.0%) | 34 (100.0%) |  |
| Antidepressants (choice=Escitalopram (Lexapro)), N (%) | 0 |  |  |  | **0.034** |
| Checked |  | 4 (5.7%) | 0 (0.0%) | 4 (11.8%) |  |
| Unchecked |  | 66 (94.3%) | 36 (100.0%) | 30 (88.2%) |  |
| Antidepressants (choice=Citalopram (Celexa)), N (%) | 0 |  |  |  |  |
| Unchecked |  | 70 (100.0%) | 36 (100.0%) | 34 (100.0%) |  |
| Antidepressants (choice=Fluvoxamine (Luvox)), N (%) | 0 |  |  |  |  |
| Unchecked |  | 70 (100.0%) | 36 (100.0%) | 34 (100.0%) |  |
| Antidepressants (choice=Venlafaxine (Effexor)), N (%) | 0 |  |  |  | 0.33 |
| Checked |  | 1 (1.4%) | 1 (2.8%) | 0 (0.0%) |  |
| Unchecked |  | 69 (98.6%) | 35 (97.2%) | 34 (100.0%) |  |
| Antidepressants (choice=Duloxetine (Cymbalta)), N (%) | 0 |  |  |  |  |
| Unchecked |  | 70 (100.0%) | 36 (100.0%) | 34 (100.0%) |  |
| Antidepressants (choice=Desvenlafaxine (Pristiq)), N (%) | 0 |  |  |  |  |
| Unchecked |  | 70 (100.0%) | 36 (100.0%) | 34 (100.0%) |  |
| Antidepressants (choice=Levomilnacipran (Fetzima)), N (%) | 0 |  |  |  |  |
| Unchecked |  | 70 (100.0%) | 36 (100.0%) | 34 (100.0%) |  |
| Antidepressants (choice=Amitriptyline (Elavil)), N (%) | 0 |  |  |  |  |
| Unchecked |  | 70 (100.0%) | 36 (100.0%) | 34 (100.0%) |  |
| Antidepressants (choice=Imipramine (Tofranil)), N (%) | 0 |  |  |  |  |
| Unchecked |  | 70 (100.0%) | 36 (100.0%) | 34 (100.0%) |  |
| Antidepressants (choice=Nortriptyline (Pamelor)), N (%) | 0 |  |  |  |  |
| Unchecked |  | 70 (100.0%) | 36 (100.0%) | 34 (100.0%) |  |
| Antidepressants (choice=Clomipramine (Anafranil)), N (%) | 0 |  |  |  |  |
| Unchecked |  | 70 (100.0%) | 36 (100.0%) | 34 (100.0%) |  |
| Antidepressants (choice=Doxepin (Sinequan)), N (%) | 0 |  |  |  |  |
| Unchecked |  | 70 (100.0%) | 36 (100.0%) | 34 (100.0%) |  |
| Antidepressants (choice=Phenelzine (Nardil)), N (%) | 0 |  |  |  |  |
| Unchecked |  | 70 (100.0%) | 36 (100.0%) | 34 (100.0%) |  |
| Antidepressants (choice=Tranylcypromine (Parnate)), N (%) | 0 |  |  |  |  |
| Unchecked |  | 70 (100.0%) | 36 (100.0%) | 34 (100.0%) |  |
| Antidepressants (choice=Isocarboxazid (Marplan)), N (%) | 0 |  |  |  |  |
| Unchecked |  | 70 (100.0%) | 36 (100.0%) | 34 (100.0%) |  |
| Antidepressants (choice=Selegiline (Emsam)), N (%) | 0 |  |  |  |  |
| Unchecked |  | 70 (100.0%) | 36 (100.0%) | 34 (100.0%) |  |
| Antidepressants (choice=Bupropion (Wellbutrin)), N (%) | 0 |  |  |  | **0.068** |
| Checked |  | 3 (4.3%) | 0 (0.0%) | 3 (8.8%) |  |
| Unchecked |  | 67 (95.7%) | 36 (100.0%) | 31 (91.2%) |  |
| Antidepressants (choice=Mirtazapine (Remeron)), N (%) | 0 |  |  |  |  |
| Unchecked |  | 70 (100.0%) | 36 (100.0%) | 34 (100.0%) |  |
| Antidepressants (choice=Trazodone (Desyrel)), N (%) | 0 |  |  |  |  |
| Unchecked |  | 70 (100.0%) | 36 (100.0%) | 34 (100.0%) |  |
| Antidepressants (choice=Vilazodone (Viibryd)), N (%) | 0 |  |  |  |  |
| Unchecked |  | 70 (100.0%) | 36 (100.0%) | 34 (100.0%) |  |
| Antidepressants (choice=Vortioxetine (Trintellix)), N (%) | 0 |  |  |  |  |
| Unchecked |  | 70 (100.0%) | 36 (100.0%) | 34 (100.0%) |  |
| Antipsychotics (choice=Aripiprazole (Abilify)), N (%) | 0 |  |  |  | 0.52 |
| Checked |  | 3 (4.3%) | 1 (2.8%) | 2 (5.9%) |  |
| Unchecked |  | 67 (95.7%) | 35 (97.2%) | 32 (94.1%) |  |
| Antipsychotics (choice=Risperidone (Risperdal)), N (%) | 0 |  |  |  | 0.75 |
| Checked |  | 7 (10.0%) | 4 (11.1%) | 3 (8.8%) |  |
| Unchecked |  | 63 (90.0%) | 32 (88.9%) | 31 (91.2%) |  |
| Antipsychotics (choice=Olanzapine (Zyprexa)), N (%) | 0 |  |  |  |  |
| Unchecked |  | 70 (100.0%) | 36 (100.0%) | 34 (100.0%) |  |
| Antipsychotics (choice=Quetiapine (Seroquel)), N (%) | 0 |  |  |  | **0.068** |
| Checked |  | 3 (4.3%) | 0 (0.0%) | 3 (8.8%) |  |
| Unchecked |  | 67 (95.7%) | 36 (100.0%) | 31 (91.2%) |  |
| Antipsychotics (choice=Ziprasidone (Geodon)), N (%) | 0 |  |  |  |  |
| Unchecked |  | 70 (100.0%) | 36 (100.0%) | 34 (100.0%) |  |
| Antipsychotics (choice=Lurasidone (Latuda)), N (%) | 0 |  |  |  |  |
| Unchecked |  | 70 (100.0%) | 36 (100.0%) | 34 (100.0%) |  |
| Antipsychotics (choice=Paliperidone (Invega)), N (%) | 0 |  |  |  |  |
| Unchecked |  | 70 (100.0%) | 36 (100.0%) | 34 (100.0%) |  |
| Antipsychotics (choice=Asenapine (Saphris)), N (%) | 0 |  |  |  |  |
| Unchecked |  | 70 (100.0%) | 36 (100.0%) | 34 (100.0%) |  |
| Antipsychotics (choice=Iloperidone (Fanapt)), N (%) | 0 |  |  |  |  |
| Unchecked |  | 70 (100.0%) | 36 (100.0%) | 34 (100.0%) |  |
| Antipsychotics (choice=Brexpiprazole (Rexulti)), N (%) | 0 |  |  |  |  |
| Unchecked |  | 70 (100.0%) | 36 (100.0%) | 34 (100.0%) |  |
| Antipsychotics (choice=Cariprazine (Vraylar)), N (%) | 0 |  |  |  |  |
| Unchecked |  | 70 (100.0%) | 36 (100.0%) | 34 (100.0%) |  |
| Antipsychotics (choice=Haloperidol (Haldol)), N (%) | 0 |  |  |  |  |
| Unchecked |  | 70 (100.0%) | 36 (100.0%) | 34 (100.0%) |  |
| Antipsychotics (choice=Fluphenazine (Prolixin)), N (%) | 0 |  |  |  |  |
| Unchecked |  | 70 (100.0%) | 36 (100.0%) | 34 (100.0%) |  |
| Antipsychotics (choice=Chlorpromazine (Thorazine)), N (%) | 0 |  |  |  |  |
| Unchecked |  | 70 (100.0%) | 36 (100.0%) | 34 (100.0%) |  |
| Antipsychotics (choice=Thioridazine (Mellaril)), N (%) | 0 |  |  |  |  |
| Unchecked |  | 70 (100.0%) | 36 (100.0%) | 34 (100.0%) |  |
| Antipsychotics (choice=Perphenazine (Trilafon)), N (%) | 0 |  |  |  |  |
| Unchecked |  | 70 (100.0%) | 36 (100.0%) | 34 (100.0%) |  |
| Antipsychotics (choice=Molindone (Moban)), N (%) | 0 |  |  |  |  |
| Unchecked |  | 70 (100.0%) | 36 (100.0%) | 34 (100.0%) |  |
| Mood Stabilizers (choice=Lithium), N (%) | 0 |  |  |  |  |
| Unchecked |  | 70 (100.0%) | 36 (100.0%) | 34 (100.0%) |  |
| Mood Stabilizers (choice=Valproate (Depakote)), N (%) | 0 |  |  |  |  |
| Unchecked |  | 70 (100.0%) | 36 (100.0%) | 34 (100.0%) |  |
| Mood Stabilizers (choice=Lamotrigine (Lamictal)), N (%) | 0 |  |  |  |  |
| Unchecked |  | 70 (100.0%) | 36 (100.0%) | 34 (100.0%) |  |
| Mood Stabilizers (choice=Carbamazepine (Tegretol)), N (%) | 0 |  |  |  |  |
| Unchecked |  | 70 (100.0%) | 36 (100.0%) | 34 (100.0%) |  |
| Mood Stabilizers (choice=Oxcarbazepine (Trileptal)), N (%) | 0 |  |  |  |  |
| Unchecked |  | 70 (100.0%) | 36 (100.0%) | 34 (100.0%) |  |
| Mood Stabilizers (choice=Topiramate (Topamax)), N (%) | 0 |  |  |  |  |
| Unchecked |  | 70 (100.0%) | 36 (100.0%) | 34 (100.0%) |  |
| Mood Stabilizers (choice=Gabapentin (Neurontin)), N (%) | 0 |  |  |  |  |
| Unchecked |  | 70 (100.0%) | 36 (100.0%) | 34 (100.0%) |  |
| Mood Stabilizers (choice=Pregabalin (Lyrica)), N (%) | 0 |  |  |  |  |
| Unchecked |  | 70 (100.0%) | 36 (100.0%) | 34 (100.0%) |  |
| Mood Stabilizers (choice=Divalproex (Depakene)), N (%) | 0 |  |  |  |  |
| Unchecked |  | 70 (100.0%) | 36 (100.0%) | 34 (100.0%) |  |
| Anxiolytics (choice=Alprazolam (Xanax)), N (%) | 0 |  |  |  |  |
| Unchecked |  | 70 (100.0%) | 36 (100.0%) | 34 (100.0%) |  |
| Anxiolytics (choice=Clonazepam (Klonopin)), N (%) | 0 |  |  |  |  |
| Unchecked |  | 70 (100.0%) | 36 (100.0%) | 34 (100.0%) |  |
| Anxiolytics (choice=Lorazepam (Ativan)), N (%) | 0 |  |  |  |  |
| Unchecked |  | 70 (100.0%) | 36 (100.0%) | 34 (100.0%) |  |
| Anxiolytics (choice=Diazepam (Valium)), N (%) | 0 |  |  |  |  |
| Unchecked |  | 70 (100.0%) | 36 (100.0%) | 34 (100.0%) |  |
| Anxiolytics (choice=Chlordiazepoxide (Librium)), N (%) | 0 |  |  |  |  |
| Unchecked |  | 70 (100.0%) | 36 (100.0%) | 34 (100.0%) |  |
| Anxiolytics (choice=Temazepam (Restoril)), N (%) | 0 |  |  |  |  |
| Unchecked |  | 70 (100.0%) | 36 (100.0%) | 34 (100.0%) |  |
| Anxiolytics (choice=Buspirone (Buspar)), N (%) | 0 |  |  |  | 0.30 |
| Checked |  | 1 (1.4%) | 0 (0.0%) | 1 (2.9%) |  |
| Unchecked |  | 69 (98.6%) | 36 (100.0%) | 33 (97.1%) |  |
| Anxiolytics (choice=Hydroxyzine (Vistaril, Atarax)), N (%) | 0 |  |  |  |  |
| Unchecked |  | 70 (100.0%) | 36 (100.0%) | 34 (100.0%) |  |
| Talk Therapy, N (%) | 0 |  |  |  | 0.23 |
| No |  | 19 (27.1%) | 8 (22.2%) | 11 (32.4%) |  |
| Unknown |  | 42 (60.0%) | 25 (69.4%) | 17 (50.0%) |  |
| Yes |  | 9 (12.9%) | 3 (8.3%) | 6 (17.6%) |  |
| Duration of Therapy, N (%) | 69 |  |  |  |  |
| 3-6 months |  | 1 (100.0%) | 1 (100.0%) |  |  |
| Type of Birth, N (%) | 0 |  |  |  | 0.33 |
| Cesarean (c-section) |  | 17 (24.3%) | 7 (19.4%) | 10 (29.4%) |  |
| Vaginal |  | 53 (75.7%) | 29 (80.6%) | 24 (70.6%) |  |
| Reason for Cesarean Section, N (%) | 53 |  |  |  | 0.39 |
| 'Cat II tracing remote from delivery' |  | 1 (5.9%) | 1 (14.3%) | 0 (0.0%) |  |
| breech presentation and cHTN w/ SI PEC w/o SF |  | 1 (5.9%) | 0 (0.0%) | 1 (10.0%) |  |
| elective |  | 1 (5.9%) | 0 (0.0%) | 1 (10.0%) |  |
| fetal bradycardia |  | 1 (5.9%) | 0 (0.0%) | 1 (10.0%) |  |
| fetal malpresentation, severe preeclampsia with severe features |  | 1 (5.9%) | 1 (14.3%) | 0 (0.0%) |  |
| fetal malpresentation, twins |  | 1 (5.9%) | 0 (0.0%) | 1 (10.0%) |  |
| hsv |  | 1 (5.9%) | 0 (0.0%) | 1 (10.0%) |  |
| hx of c-section |  | 1 (5.9%) | 0 (0.0%) | 1 (10.0%) |  |
| hx of shoulder dystocia |  | 1 (5.9%) | 1 (14.3%) | 0 (0.0%) |  |
| hysterotomy |  | 1 (5.9%) | 1 (14.3%) | 0 (0.0%) |  |
| non-assuring FHTs |  | 1 (5.9%) | 1 (14.3%) | 0 (0.0%) |  |
| non-reassuring fhts |  | 1 (5.9%) | 0 (0.0%) | 1 (10.0%) |  |
| preeclampsia |  | 1 (5.9%) | 0 (0.0%) | 1 (10.0%) |  |
| previous C-sections |  | 1 (5.9%) | 0 (0.0%) | 1 (10.0%) |  |
| prior c-section |  | 1 (5.9%) | 0 (0.0%) | 1 (10.0%) |  |
| repeat |  | 1 (5.9%) | 1 (14.3%) | 0 (0.0%) |  |
| terminal fetal bradycardia |  | 1 (5.9%) | 1 (14.3%) | 0 (0.0%) |  |
| NICU admission? (Gestation Age < 35 weeks), N (%) | 0 |  |  |  | **0.085** |
| No |  | 67 (95.7%) | 33 (91.7%) | 34 (100.0%) |  |
| Yes |  | 3 (4.3%) | 3 (8.3%) | 0 (0.0%) |  |
| Postpartum Hemorrhage? (< /= 1000 mL at delivery), N (%) | 0 |  |  |  | 0.28 |
| No |  | 66 (94.3%) | 35 (97.2%) | 31 (91.2%) |  |
| Yes |  | 4 (5.7%) | 1 (2.8%) | 3 (8.8%) |  |
| Severe Preeclampsia?, N (%) | 0 |  |  |  | 0.24 |
| No |  | 61 (87.1%) | 33 (91.7%) | 28 (82.4%) |  |
| Yes |  | 9 (12.9%) | 3 (8.3%) | 6 (17.6%) |  |
| Complete?, N (%) | 0 |  |  |  |  |
| Complete |  | 70 (100.0%) | 36 (100.0%) | 34 (100.0%) |  |
| prenatal EPDS score1, N | 17 | 53 | 23 | 30 | 0.76 |
| Mean (SD) |  | 12.3 (6.4) | 12.6 (6.3) | 12.1 (6.5) |  |
| Median (IQR) |  | 12.0 (8.0, 17.0) | 12.0 (10.0, 17.0) | 11.5 (7.0, 18.0) |  |
| Median (Range) |  | 12.0 (0.0, 25.0) | 12.0 (0.0, 23.0) | 11.5 (1.0, 25.0) |  |
| postpartum EPDS score1, N | 37 | 33 | 0 | 33 |  |
| Mean (SD) |  | 7.5 (5.5) | . (.) | 7.5 (5.5) |  |
| Median (IQR) |  | 7.0 (3.0, 12.0) | . (., .) | 7.0 (3.0, 12.0) |  |
| Median (Range) |  | 7.0 (0.0, 19.0) | . (., .) | 7.0 (0.0, 19.0) |  |
| lost to follow up?, N (%) | 0 |  |  |  | **<.0001** |
| no |  | 34 (48.6%) | 0 (0.0%) | 34 (100.0%) |  |
| yes |  | 36 (51.4%) | 36 (100.0%) | 0 (0.0%) |  |

|  | | | **When Referred?** | |  |
| --- | --- | --- | --- | --- | --- |
| **Variable** | **NMiss** | **Overall (n=61)** | **Before birth (n=36)** | **After birth (n=25)** | **Chisq or T-test p-value** |
| Have seen Dr. Tabi, N (%) | 0 |  |  |  | **0.039** |
| No |  | 18 (29.5%) | 7 (19.4%) | 11 (44.0%) |  |
| Yes |  | 43 (70.5%) | 29 (80.6%) | 14 (56.0%) |  |
| Prenatal EPDS SI, N (%) | 19 |  |  |  | 0.59 |
| Hardly ever |  | 5 (11.9%) | 3 (11.1%) | 2 (13.3%) |  |
| Never/Denies |  | 33 (78.6%) | 22 (81.5%) | 11 (73.3%) |  |
| Sometimes |  | 3 (7.1%) | 2 (7.4%) | 1 (6.7%) |  |
| Yes, quite often |  | 1 (2.4%) | 0 (0.0%) | 1 (6.7%) |  |
| Postpartum EPDS SI, N (%) | 34 |  |  |  |  |
| Never/Denies |  | 27 (100.0%) | 15 (100.0%) | 12 (100.0%) |  |
| Age, N | 1 | 60 | 35 | 25 | 0.66 |
| Mean (SD) |  | 27.7 (6.1) | 27.4 (6.4) | 28.1 (5.8) |  |
| Median (IQR) |  | 27.5 (22.0, 32.0) | 26.0 (21.0, 32.0) | 28.0 (25.0, 32.0) |  |
| Median (Range) |  | 27.5 (17.0, 39.0) | 26.0 (18.0, 39.0) | 28.0 (17.0, 38.0) |  |
| Ethnicity (choice=Hispanic/Latino of any race), N (%) | 0 |  |  |  | 0.51 |
| Checked |  | 20 (32.8%) | 13 (36.1%) | 7 (28.0%) |  |
| Unchecked |  | 41 (67.2%) | 23 (63.9%) | 18 (72.0%) |  |
| Ethnicity (choice=American Indian or Alaska Native), N (%) | 0 |  |  |  |  |
| Unchecked |  | 61 (100.0%) | 36 (100.0%) | 25 (100.0%) |  |
| Ethnicity (choice=Asian), N (%) | 0 |  |  |  |  |
| Unchecked |  | 61 (100.0%) | 36 (100.0%) | 25 (100.0%) |  |
| Ethnicity (choice=Black or African American), N (%) | 0 |  |  |  | 0.95 |
| Checked |  | 29 (47.5%) | 17 (47.2%) | 12 (48.0%) |  |
| Unchecked |  | 32 (52.5%) | 19 (52.8%) | 13 (52.0%) |  |
| Ethnicity (choice=Native Hawaiian or Other Pacific Islander), N (%) | 0 |  |  |  |  |
| Unchecked |  | 61 (100.0%) | 36 (100.0%) | 25 (100.0%) |  |
| Ethnicity (choice=White), N (%) | 0 |  |  |  | 0.74 |
| Checked |  | 11 (18.0%) | 6 (16.7%) | 5 (20.0%) |  |
| Unchecked |  | 50 (82.0%) | 30 (83.3%) | 20 (80.0%) |  |
| Ethnicity (choice=Unknown), N (%) | 0 |  |  |  | 0.79 |
| Checked |  | 2 (3.3%) | 1 (2.8%) | 1 (4.0%) |  |
| Unchecked |  | 59 (96.7%) | 35 (97.2%) | 24 (96.0%) |  |
| Employed?, N (%) | 0 |  |  |  | 0.29 |
| No |  | 33 (54.1%) | 22 (61.1%) | 11 (44.0%) |  |
| Unknown |  | 8 (13.1%) | 3 (8.3%) | 5 (20.0%) |  |
| Yes |  | 20 (32.8%) | 11 (30.6%) | 9 (36.0%) |  |
| Unhoused?, N (%) | 0 |  |  |  | 0.37 |
| No |  | 56 (91.8%) | 34 (94.4%) | 22 (88.0%) |  |
| Unknown |  | 5 (8.2%) | 2 (5.6%) | 3 (12.0%) |  |
| Substance use?, N (%) | 0 |  |  |  | 0.19 |
| No |  | 19 (31.1%) | 11 (30.6%) | 8 (32.0%) |  |
| Unknown |  | 11 (18.0%) | 4 (11.1%) | 7 (28.0%) |  |
| Yes |  | 31 (50.8%) | 21 (58.3%) | 10 (40.0%) |  |
| Substance use during pregnancy?, N (%) | 30 |  |  |  | **0.093** |
| No |  | 12 (38.7%) | 6 (28.6%) | 6 (60.0%) |  |
| Yes |  | 19 (61.3%) | 15 (71.4%) | 4 (40.0%) |  |
| Substance Type? (choice=Alcohol), N (%) | 0 |  |  |  | 0.53 |
| Checked |  | 10 (16.4%) | 5 (13.9%) | 5 (20.0%) |  |
| Unchecked |  | 51 (83.6%) | 31 (86.1%) | 20 (80.0%) |  |
| Substance Type? (choice=Caffeine), N (%) | 0 |  |  |  | 0.40 |
| Checked |  | 1 (1.6%) | 1 (2.8%) | 0 (0.0%) |  |
| Unchecked |  | 60 (98.4%) | 35 (97.2%) | 25 (100.0%) |  |
| Substance Type? (choice=Cannabis (Marijuana)), N (%) | 0 |  |  |  | **0.017** |
| Checked |  | 23 (37.7%) | 18 (50.0%) | 5 (20.0%) |  |
| Unchecked |  | 38 (62.3%) | 18 (50.0%) | 20 (80.0%) |  |
| Substance Type? (choice=LSD (Lysergic Acid Diethylamide)), N (%) | 0 |  |  |  |  |
| Unchecked |  | 61 (100.0%) | 36 (100.0%) | 25 (100.0%) |  |
| Substance Type? (choice=Psilocybin (Magic Mushrooms)), N (%) | 0 |  |  |  |  |
| Unchecked |  | 61 (100.0%) | 36 (100.0%) | 25 (100.0%) |  |
| Substance Type? (choice=Phencyclidine (PCP)), N (%) | 0 |  |  |  | 0.40 |
| Checked |  | 1 (1.6%) | 1 (2.8%) | 0 (0.0%) |  |
| Unchecked |  | 60 (98.4%) | 35 (97.2%) | 25 (100.0%) |  |
| Substance Type? (choice=Ketamine), N (%) | 0 |  |  |  |  |
| Unchecked |  | 61 (100.0%) | 36 (100.0%) | 25 (100.0%) |  |
| Substance Type? (choice=Inhalants (e.g., paint thinner, gasoline fumes)), N (%) | 0 |  |  |  |  |
| Unchecked |  | 61 (100.0%) | 36 (100.0%) | 25 (100.0%) |  |
| Substance Type? (choice=Opioids (e.g., heroin, prescription pain medications)), N (%) | 0 |  |  |  | 0.20 |
| Checked |  | 6 (9.8%) | 5 (13.9%) | 1 (4.0%) |  |
| Unchecked |  | 55 (90.2%) | 31 (86.1%) | 24 (96.0%) |  |
| Substance Type? (choice=Benzodiazepines (e.g., Xanax, Valium)), N (%) | 0 |  |  |  | 0.70 |
| Checked |  | 4 (6.6%) | 2 (5.6%) | 2 (8.0%) |  |
| Unchecked |  | 57 (93.4%) | 34 (94.4%) | 23 (92.0%) |  |
| Substance Type? (choice=Barbiturates (e.g., Phenobarbital)), N (%) | 0 |  |  |  |  |
| Unchecked |  | 61 (100.0%) | 36 (100.0%) | 25 (100.0%) |  |
| Substance Type? (choice=Non-Benzodiazepine Hypnotics (e.g., Ambien, Lunesta)), N (%) | 0 |  |  |  |  |
| Unchecked |  | 61 (100.0%) | 36 (100.0%) | 25 (100.0%) |  |
| Substance Type? (choice=Amphetamines (e.g., Adderall, methamphetamine)), N (%) | 0 |  |  |  | 0.23 |
| Checked |  | 2 (3.3%) | 2 (5.6%) | 0 (0.0%) |  |
| Unchecked |  | 59 (96.7%) | 34 (94.4%) | 25 (100.0%) |  |
| Substance Type? (choice=Cocaine), N (%) | 0 |  |  |  | 0.64 |
| Checked |  | 6 (9.8%) | 3 (8.3%) | 3 (12.0%) |  |
| Unchecked |  | 55 (90.2%) | 33 (91.7%) | 22 (88.0%) |  |
| Substance Type? (choice=Tobacco (Nicotine)), N (%) | 0 |  |  |  | 0.32 |
| Checked |  | 8 (13.1%) | 6 (16.7%) | 2 (8.0%) |  |
| Unchecked |  | 53 (86.9%) | 30 (83.3%) | 23 (92.0%) |  |
| Substance Type? (choice=Synthetic Cannabinoids (e.g., Spice, K2)), N (%) | 0 |  |  |  |  |
| Unchecked |  | 61 (100.0%) | 36 (100.0%) | 25 (100.0%) |  |
| Substance Type? (choice=Synthetic Cathinones (e.g., Bath Salts)), N (%) | 0 |  |  |  |  |
| Unchecked |  | 61 (100.0%) | 36 (100.0%) | 25 (100.0%) |  |
| Substance Type? (choice=Other or Unknown Substances), N (%) | 0 |  |  |  |  |
| Unchecked |  | 61 (100.0%) | 36 (100.0%) | 25 (100.0%) |  |
| Method of Substance Use? (choice=Inhalation (e.g., smoking, vaping)), N (%) | 0 |  |  |  | 0.40 |
| Checked |  | 1 (1.6%) | 1 (2.8%) | 0 (0.0%) |  |
| Unchecked |  | 60 (98.4%) | 35 (97.2%) | 25 (100.0%) |  |
| Method of Substance Use? (choice=Oral Ingestion (e.g., swallowing pills or liquids)), N (%) | 0 |  |  |  |  |
| Unchecked |  | 61 (100.0%) | 36 (100.0%) | 25 (100.0%) |  |
| Method of Substance Use? (choice=Intranasal (e.g., snorting or sniffing)), N (%) | 0 |  |  |  |  |
| Unchecked |  | 61 (100.0%) | 36 (100.0%) | 25 (100.0%) |  |
| Method of Substance Use? (choice=Intravenous (IV) Injection), N (%) | 0 |  |  |  |  |
| Unchecked |  | 61 (100.0%) | 36 (100.0%) | 25 (100.0%) |  |
| Method of Substance Use? (choice=Intramuscular (IM) Injection), N (%) | 0 |  |  |  |  |
| Unchecked |  | 61 (100.0%) | 36 (100.0%) | 25 (100.0%) |  |
| Method of Substance Use? (choice=Subcutaneous (SC) Injection), N (%) | 0 |  |  |  |  |
| Unchecked |  | 61 (100.0%) | 36 (100.0%) | 25 (100.0%) |  |
| Method of Substance Use? (choice=Transdermal (e.g., patches)), N (%) | 0 |  |  |  |  |
| Unchecked |  | 61 (100.0%) | 36 (100.0%) | 25 (100.0%) |  |
| Method of Substance Use? (choice=Rectal (e.g., suppositories)), N (%) | 0 |  |  |  |  |
| Unchecked |  | 61 (100.0%) | 36 (100.0%) | 25 (100.0%) |  |
| Method of Substance Use? (choice=Sublingual (under the tongue)), N (%) | 0 |  |  |  |  |
| Unchecked |  | 61 (100.0%) | 36 (100.0%) | 25 (100.0%) |  |
| Method of Substance Use? (choice=Transbuccal (between the cheek and gum)), N (%) | 0 |  |  |  |  |
| Unchecked |  | 61 (100.0%) | 36 (100.0%) | 25 (100.0%) |  |
| History of abuse?, N (%) | 0 |  |  |  | 0.95 |
| No |  | 15 (24.6%) | 9 (25.0%) | 6 (24.0%) |  |
| Unknown |  | 23 (37.7%) | 13 (36.1%) | 10 (40.0%) |  |
| Yes |  | 23 (37.7%) | 14 (38.9%) | 9 (36.0%) |  |
| Relationship Status, N (%) | 0 |  |  |  | 0.49 |
| Divorced |  | 1 (1.6%) | 0 (0.0%) | 1 (4.0%) |  |
| Has long-term partner |  | 32 (52.5%) | 20 (55.6%) | 12 (48.0%) |  |
| Married |  | 5 (8.2%) | 3 (8.3%) | 2 (8.0%) |  |
| Single |  | 8 (13.1%) | 6 (16.7%) | 2 (8.0%) |  |
| Unknown |  | 15 (24.6%) | 7 (19.4%) | 8 (32.0%) |  |
| Education Level, N (%) | 0 |  |  |  | 0.26 |
| Associates degree |  | 2 (3.3%) | 1 (2.8%) | 1 (4.0%) |  |
| Bachelors degree |  | 1 (1.6%) | 0 (0.0%) | 1 (4.0%) |  |
| Below High School |  | 9 (14.8%) | 7 (19.4%) | 2 (8.0%) |  |
| Graduate Degree |  | 2 (3.3%) | 0 (0.0%) | 2 (8.0%) |  |
| High School |  | 16 (26.2%) | 11 (30.6%) | 5 (20.0%) |  |
| Unknown |  | 31 (50.8%) | 17 (47.2%) | 14 (56.0%) |  |
| Previous IP hospitalizations?, N (%) | 1 |  |  |  | 0.19 |
| Found |  | 12 (20.0%) | 9 (25.7%) | 3 (12.0%) |  |
| Not Found |  | 48 (80.0%) | 26 (74.3%) | 22 (88.0%) |  |
| Psychiatric Diagnosis (choice=None), N (%) | 0 |  |  |  | 0.43 |
| Checked |  | 14 (23.0%) | 7 (19.4%) | 7 (28.0%) |  |
| Unchecked |  | 47 (77.0%) | 29 (80.6%) | 18 (72.0%) |  |
| Psychiatric Diagnosis (choice=Bipolar Disorders), N (%) | 0 |  |  |  | **0.007** |
| Checked |  | 9 (14.8%) | 9 (25.0%) | 0 (0.0%) |  |
| Unchecked |  | 52 (85.2%) | 27 (75.0%) | 25 (100.0%) |  |
| Psychiatric Diagnosis (choice=Major Depressive Disorder (MDD)), N (%) | 0 |  |  |  | 0.58 |
| Checked |  | 27 (44.3%) | 17 (47.2%) | 10 (40.0%) |  |
| Unchecked |  | 34 (55.7%) | 19 (52.8%) | 15 (60.0%) |  |
| Psychiatric Diagnosis (choice=Schizophrenia and Other Psychotic Disorders), N (%) | 0 |  |  |  | **0.052** |
| Checked |  | 5 (8.2%) | 5 (13.9%) | 0 (0.0%) |  |
| Unchecked |  | 56 (91.8%) | 31 (86.1%) | 25 (100.0%) |  |
| Psychiatric Diagnosis (choice=Generalized Anxiety Disorder (GAD)), N (%) | 0 |  |  |  | 0.93 |
| Checked |  | 24 (39.3%) | 14 (38.9%) | 10 (40.0%) |  |
| Unchecked |  | 37 (60.7%) | 22 (61.1%) | 15 (60.0%) |  |
| Psychiatric Diagnosis (choice=Borderline Personality Disorder), N (%) | 0 |  |  |  | 0.23 |
| Checked |  | 1 (1.6%) | 0 (0.0%) | 1 (4.0%) |  |
| Unchecked |  | 60 (98.4%) | 36 (100.0%) | 24 (96.0%) |  |
| Psychiatric Diagnosis (choice=Substance Use Disorder), N (%) | 0 |  |  |  | 0.58 |
| Checked |  | 8 (13.1%) | 4 (11.1%) | 4 (16.0%) |  |
| Unchecked |  | 53 (86.9%) | 32 (88.9%) | 21 (84.0%) |  |
| Psychiatric Diagnosis (choice=Obsessive Compulsive Disorder (OCD)), N (%) | 0 |  |  |  |  |
| Unchecked |  | 61 (100.0%) | 36 (100.0%) | 25 (100.0%) |  |
| Psychiatric Diagnosis (choice=Posttraumatic Stress Disorder (PTSD)), N (%) | 0 |  |  |  | 0.51 |
| Checked |  | 20 (32.8%) | 13 (36.1%) | 7 (28.0%) |  |
| Unchecked |  | 41 (67.2%) | 23 (63.9%) | 18 (72.0%) |  |
| Psychiatric Diagnosis (choice=postpartum depressive disorder), N (%) | 0 |  |  |  | **0.011** |
| Checked |  | 7 (11.5%) | 1 (2.8%) | 6 (24.0%) |  |
| Unchecked |  | 54 (88.5%) | 35 (97.2%) | 19 (76.0%) |  |
| Diagnoser, N (%) | 13 |  |  |  | 0.65 |
| Dr. Tabi |  | 33 (68.8%) | 22 (73.3%) | 11 (61.1%) |  |
| Other |  | 7 (14.6%) | 4 (13.3%) | 3 (16.7%) |  |
| Self-reported |  | 8 (16.7%) | 4 (13.3%) | 4 (22.2%) |  |
| Bipolar Disorders (choice=Bipolar I Disorder), N (%) | 0 |  |  |  | 0.23 |
| Checked |  | 2 (3.3%) | 2 (5.6%) | 0 (0.0%) |  |
| Unchecked |  | 59 (96.7%) | 34 (94.4%) | 25 (100.0%) |  |
| Bipolar Disorders (choice=Bipolar II Disorder), N (%) | 0 |  |  |  | 0.40 |
| Checked |  | 1 (1.6%) | 1 (2.8%) | 0 (0.0%) |  |
| Unchecked |  | 60 (98.4%) | 35 (97.2%) | 25 (100.0%) |  |
| Bipolar Disorders (choice=Cyclothymic Disorder (Cyclothymia)), N (%) | 0 |  |  |  |  |
| Unchecked |  | 61 (100.0%) | 36 (100.0%) | 25 (100.0%) |  |
| Bipolar Disorders (choice=Other Specified Bipolar and Related Disorder), N (%) | 0 |  |  |  | 0.40 |
| Checked |  | 1 (1.6%) | 1 (2.8%) | 0 (0.0%) |  |
| Unchecked |  | 60 (98.4%) | 35 (97.2%) | 25 (100.0%) |  |
| Bipolar Disorders (choice=Unspecified Bipolar and Related Disorder), N (%) | 0 |  |  |  | 0.23 |
| Checked |  | 2 (3.3%) | 2 (5.6%) | 0 (0.0%) |  |
| Unchecked |  | 59 (96.7%) | 34 (94.4%) | 25 (100.0%) |  |
| Substance Use Disorders (per DSM5) (choice=Alcohol Use Disorder), N (%) | 0 |  |  |  | 0.23 |
| Checked |  | 1 (1.6%) | 0 (0.0%) | 1 (4.0%) |  |
| Unchecked |  | 60 (98.4%) | 36 (100.0%) | 24 (96.0%) |  |
| Substance Use Disorders (per DSM5) (choice=Caffeine Use Disorder), N (%) | 0 |  |  |  |  |
| Unchecked |  | 61 (100.0%) | 36 (100.0%) | 25 (100.0%) |  |
| Substance Use Disorders (per DSM5) (choice=Cannabis Use Disorder), N (%) | 0 |  |  |  | 0.78 |
| Checked |  | 3 (4.9%) | 2 (5.6%) | 1 (4.0%) |  |
| Unchecked |  | 58 (95.1%) | 34 (94.4%) | 24 (96.0%) |  |
| Substance Use Disorders (per DSM5) (choice=Hallucinogen Use Disorder), N (%) | 0 |  |  |  | 0.40 |
| Checked |  | 1 (1.6%) | 1 (2.8%) | 0 (0.0%) |  |
| Unchecked |  | 60 (98.4%) | 35 (97.2%) | 25 (100.0%) |  |
| Substance Use Disorders (per DSM5) (choice=Inhalant Use Disorder), N (%) | 0 |  |  |  |  |
| Unchecked |  | 61 (100.0%) | 36 (100.0%) | 25 (100.0%) |  |
| Substance Use Disorders (per DSM5) (choice=Opioid Use Disorder), N (%) | 0 |  |  |  | 0.79 |
| Checked |  | 2 (3.3%) | 1 (2.8%) | 1 (4.0%) |  |
| Unchecked |  | 59 (96.7%) | 35 (97.2%) | 24 (96.0%) |  |
| Substance Use Disorders (per DSM5) (choice=Sedative, Hypnotic, or Anxiolytic Use Disorder), N (%) | 0 |  |  |  | 0.23 |
| Checked |  | 1 (1.6%) | 0 (0.0%) | 1 (4.0%) |  |
| Unchecked |  | 60 (98.4%) | 36 (100.0%) | 24 (96.0%) |  |
| Substance Use Disorders (per DSM5) (choice=Stimulant Use Disorder), N (%) | 0 |  |  |  | 0.79 |
| Checked |  | 2 (3.3%) | 1 (2.8%) | 1 (4.0%) |  |
| Unchecked |  | 59 (96.7%) | 35 (97.2%) | 24 (96.0%) |  |
| Substance Use Disorders (per DSM5) (choice=Tobacco Use Disorder), N (%) | 0 |  |  |  | 0.78 |
| Checked |  | 3 (4.9%) | 2 (5.6%) | 1 (4.0%) |  |
| Unchecked |  | 58 (95.1%) | 34 (94.4%) | 24 (96.0%) |  |
| Substance Use Disorders (per DSM5) (choice=Other (or Unknown) Substance Use Disorder), N (%) | 0 |  |  |  |  |
| Unchecked |  | 61 (100.0%) | 36 (100.0%) | 25 (100.0%) |  |
| Substance Use Disorders (per DSM5) (choice=Alcohol Intoxication), N (%) | 0 |  |  |  |  |
| Unchecked |  | 61 (100.0%) | 36 (100.0%) | 25 (100.0%) |  |
| Substance Use Disorders (per DSM5) (choice=Caffeine Intoxication), N (%) | 0 |  |  |  |  |
| Unchecked |  | 61 (100.0%) | 36 (100.0%) | 25 (100.0%) |  |
| Substance Use Disorders (per DSM5) (choice=Cannabis Intoxication), N (%) | 0 |  |  |  |  |
| Unchecked |  | 61 (100.0%) | 36 (100.0%) | 25 (100.0%) |  |
| Substance Use Disorders (per DSM5) (choice=Stimulant Intoxication (e.g., amphetamines, cocaine)), N (%) | 0 |  |  |  |  |
| Unchecked |  | 61 (100.0%) | 36 (100.0%) | 25 (100.0%) |  |
| Substance Use Disorders (per DSM5) (choice=Opioid Intoxication), N (%) | 0 |  |  |  |  |
| Unchecked |  | 61 (100.0%) | 36 (100.0%) | 25 (100.0%) |  |
| Substance Use Disorders (per DSM5) (choice=Sedative, Hypnotic, or Anxiolytic Intoxication), N (%) | 0 |  |  |  |  |
| Unchecked |  | 61 (100.0%) | 36 (100.0%) | 25 (100.0%) |  |
| Substance Use Disorders (per DSM5) (choice=Hallucinogen Intoxication), N (%) | 0 |  |  |  |  |
| Unchecked |  | 61 (100.0%) | 36 (100.0%) | 25 (100.0%) |  |
| Substance Use Disorders (per DSM5) (choice=Inhalant Intoxication), N (%) | 0 |  |  |  |  |
| Unchecked |  | 61 (100.0%) | 36 (100.0%) | 25 (100.0%) |  |
| Substance Use Disorders (per DSM5) (choice=Caffeine Intoxication), N (%) | 0 |  |  |  |  |
| Unchecked |  | 61 (100.0%) | 36 (100.0%) | 25 (100.0%) |  |
| Substance Use Disorders (per DSM5) (choice=Other (or Unknown) Substance Intoxication), N (%) | 0 |  |  |  |  |
| Unchecked |  | 61 (100.0%) | 36 (100.0%) | 25 (100.0%) |  |
| Substance Use Disorders (per DSM5) (choice=Alcohol Withdrawal), N (%) | 0 |  |  |  |  |
| Unchecked |  | 61 (100.0%) | 36 (100.0%) | 25 (100.0%) |  |
| Substance Use Disorders (per DSM5) (choice=Caffeine Withdrawal), N (%) | 0 |  |  |  |  |
| Unchecked |  | 61 (100.0%) | 36 (100.0%) | 25 (100.0%) |  |
| Substance Use Disorders (per DSM5) (choice=Cannabis Withdrawal), N (%) | 0 |  |  |  |  |
| Unchecked |  | 61 (100.0%) | 36 (100.0%) | 25 (100.0%) |  |
| Substance Use Disorders (per DSM5) (choice=Stimulant Withdrawal (e.g., amphetamines, cocaine)), N (%) | 0 |  |  |  |  |
| Unchecked |  | 61 (100.0%) | 36 (100.0%) | 25 (100.0%) |  |
| Substance Use Disorders (per DSM5) (choice=Opioid Withdrawal), N (%) | 0 |  |  |  |  |
| Unchecked |  | 61 (100.0%) | 36 (100.0%) | 25 (100.0%) |  |
| Substance Use Disorders (per DSM5) (choice=Sedative, Hypnotic, or Anxiolytic Withdrawal), N (%) | 0 |  |  |  |  |
| Unchecked |  | 61 (100.0%) | 36 (100.0%) | 25 (100.0%) |  |
| Substance Use Disorders (per DSM5) (choice=Hallucinogen Persisting Perception Disorder (not strictly withdrawal but related)), N (%) | 0 |  |  |  |  |
| Unchecked |  | 61 (100.0%) | 36 (100.0%) | 25 (100.0%) |  |
| Substance Use Disorders (per DSM5) (choice=Inhalant Withdrawal), N (%) | 0 |  |  |  |  |
| Unchecked |  | 61 (100.0%) | 36 (100.0%) | 25 (100.0%) |  |
| Substance Use Disorders (per DSM5) (choice=Caffeine Withdrawal), N (%) | 0 |  |  |  |  |
| Unchecked |  | 61 (100.0%) | 36 (100.0%) | 25 (100.0%) |  |
| Substance Use Disorders (per DSM5) (choice=Other (or Unknown) Substance Withdrawal), N (%) | 0 |  |  |  |  |
| Unchecked |  | 61 (100.0%) | 36 (100.0%) | 25 (100.0%) |  |
| Psychotropic Medication (choice=None), N (%) | 0 |  |  |  | 0.31 |
| Checked |  | 27 (44.3%) | 14 (38.9%) | 13 (52.0%) |  |
| Unchecked |  | 34 (55.7%) | 22 (61.1%) | 12 (48.0%) |  |
| Psychotropic Medication (choice=Antidepressant (e.g., SSRI, SNRI, TCA, MAOIs, etc)), N (%) | 0 |  |  |  | 0.83 |
| Checked |  | 21 (34.4%) | 12 (33.3%) | 9 (36.0%) |  |
| Unchecked |  | 40 (65.6%) | 24 (66.7%) | 16 (64.0%) |  |
| Psychotropic Medication (choice=Antipsychotic (e.g., FGA, SGA)), N (%) | 0 |  |  |  | 0.14 |
| Checked |  | 13 (21.3%) | 10 (27.8%) | 3 (12.0%) |  |
| Unchecked |  | 48 (78.7%) | 26 (72.2%) | 22 (88.0%) |  |
| Psychotropic Medication (choice=Mood Stabilizer (e.g., Lithium, AEDs)), N (%) | 0 |  |  |  |  |
| Unchecked |  | 61 (100.0%) | 36 (100.0%) | 25 (100.0%) |  |
| Psychotropic Medication (choice=Anxiolytic (e.g., Benzos, Atarax, Buspirone)), N (%) | 0 |  |  |  | 0.79 |
| Checked |  | 2 (3.3%) | 1 (2.8%) | 1 (4.0%) |  |
| Unchecked |  | 59 (96.7%) | 35 (97.2%) | 24 (96.0%) |  |
| Psychotropic Medication (choice=Sedative (e.g., Ambien)), N (%) | 0 |  |  |  | 0.23 |
| Checked |  | 1 (1.6%) | 0 (0.0%) | 1 (4.0%) |  |
| Unchecked |  | 60 (98.4%) | 36 (100.0%) | 24 (96.0%) |  |
| Psychotropic Medication (choice=Stimulant), N (%) | 0 |  |  |  | 0.79 |
| Checked |  | 2 (3.3%) | 1 (2.8%) | 1 (4.0%) |  |
| Unchecked |  | 59 (96.7%) | 35 (97.2%) | 24 (96.0%) |  |
| Antidepressants (choice=Fluoxetine (Prozac)), N (%) | 0 |  |  |  | 0.78 |
| Checked |  | 3 (4.9%) | 2 (5.6%) | 1 (4.0%) |  |
| Unchecked |  | 58 (95.1%) | 34 (94.4%) | 24 (96.0%) |  |
| Antidepressants (choice=Sertraline (Zoloft)), N (%) | 0 |  |  |  | 0.73 |
| Checked |  | 11 (18.0%) | 7 (19.4%) | 4 (16.0%) |  |
| Unchecked |  | 50 (82.0%) | 29 (80.6%) | 21 (84.0%) |  |
| Antidepressants (choice=Paroxetine (Paxil)), N (%) | 0 |  |  |  |  |
| Unchecked |  | 61 (100.0%) | 36 (100.0%) | 25 (100.0%) |  |
| Antidepressants (choice=Escitalopram (Lexapro)), N (%) | 0 |  |  |  | 0.35 |
| Checked |  | 3 (4.9%) | 1 (2.8%) | 2 (8.0%) |  |
| Unchecked |  | 58 (95.1%) | 35 (97.2%) | 23 (92.0%) |  |
| Antidepressants (choice=Citalopram (Celexa)), N (%) | 0 |  |  |  |  |
| Unchecked |  | 61 (100.0%) | 36 (100.0%) | 25 (100.0%) |  |
| Antidepressants (choice=Fluvoxamine (Luvox)), N (%) | 0 |  |  |  |  |
| Unchecked |  | 61 (100.0%) | 36 (100.0%) | 25 (100.0%) |  |
| Antidepressants (choice=Venlafaxine (Effexor)), N (%) | 0 |  |  |  | 0.23 |
| Checked |  | 1 (1.6%) | 0 (0.0%) | 1 (4.0%) |  |
| Unchecked |  | 60 (98.4%) | 36 (100.0%) | 24 (96.0%) |  |
| Antidepressants (choice=Duloxetine (Cymbalta)), N (%) | 0 |  |  |  |  |
| Unchecked |  | 61 (100.0%) | 36 (100.0%) | 25 (100.0%) |  |
| Antidepressants (choice=Desvenlafaxine (Pristiq)), N (%) | 0 |  |  |  |  |
| Unchecked |  | 61 (100.0%) | 36 (100.0%) | 25 (100.0%) |  |
| Antidepressants (choice=Levomilnacipran (Fetzima)), N (%) | 0 |  |  |  |  |
| Unchecked |  | 61 (100.0%) | 36 (100.0%) | 25 (100.0%) |  |
| Antidepressants (choice=Amitriptyline (Elavil)), N (%) | 0 |  |  |  |  |
| Unchecked |  | 61 (100.0%) | 36 (100.0%) | 25 (100.0%) |  |
| Antidepressants (choice=Imipramine (Tofranil)), N (%) | 0 |  |  |  |  |
| Unchecked |  | 61 (100.0%) | 36 (100.0%) | 25 (100.0%) |  |
| Antidepressants (choice=Nortriptyline (Pamelor)), N (%) | 0 |  |  |  |  |
| Unchecked |  | 61 (100.0%) | 36 (100.0%) | 25 (100.0%) |  |
| Antidepressants (choice=Clomipramine (Anafranil)), N (%) | 0 |  |  |  |  |
| Unchecked |  | 61 (100.0%) | 36 (100.0%) | 25 (100.0%) |  |
| Antidepressants (choice=Doxepin (Sinequan)), N (%) | 0 |  |  |  |  |
| Unchecked |  | 61 (100.0%) | 36 (100.0%) | 25 (100.0%) |  |
| Antidepressants (choice=Phenelzine (Nardil)), N (%) | 0 |  |  |  |  |
| Unchecked |  | 61 (100.0%) | 36 (100.0%) | 25 (100.0%) |  |
| Antidepressants (choice=Tranylcypromine (Parnate)), N (%) | 0 |  |  |  |  |
| Unchecked |  | 61 (100.0%) | 36 (100.0%) | 25 (100.0%) |  |
| Antidepressants (choice=Isocarboxazid (Marplan)), N (%) | 0 |  |  |  |  |
| Unchecked |  | 61 (100.0%) | 36 (100.0%) | 25 (100.0%) |  |
| Antidepressants (choice=Selegiline (Emsam)), N (%) | 0 |  |  |  |  |
| Unchecked |  | 61 (100.0%) | 36 (100.0%) | 25 (100.0%) |  |
| Antidepressants (choice=Bupropion (Wellbutrin)), N (%) | 0 |  |  |  | 0.78 |
| Checked |  | 3 (4.9%) | 2 (5.6%) | 1 (4.0%) |  |
| Unchecked |  | 58 (95.1%) | 34 (94.4%) | 24 (96.0%) |  |
| Antidepressants (choice=Mirtazapine (Remeron)), N (%) | 0 |  |  |  |  |
| Unchecked |  | 61 (100.0%) | 36 (100.0%) | 25 (100.0%) |  |
| Antidepressants (choice=Trazodone (Desyrel)), N (%) | 0 |  |  |  |  |
| Unchecked |  | 61 (100.0%) | 36 (100.0%) | 25 (100.0%) |  |
| Antidepressants (choice=Vilazodone (Viibryd)), N (%) | 0 |  |  |  |  |
| Unchecked |  | 61 (100.0%) | 36 (100.0%) | 25 (100.0%) |  |
| Antidepressants (choice=Vortioxetine (Trintellix)), N (%) | 0 |  |  |  |  |
| Unchecked |  | 61 (100.0%) | 36 (100.0%) | 25 (100.0%) |  |
| Antipsychotics (choice=Aripiprazole (Abilify)), N (%) | 0 |  |  |  | 0.35 |
| Checked |  | 3 (4.9%) | 1 (2.8%) | 2 (8.0%) |  |
| Unchecked |  | 58 (95.1%) | 35 (97.2%) | 23 (92.0%) |  |
| Antipsychotics (choice=Risperidone (Risperdal)), N (%) | 0 |  |  |  | 0.13 |
| Checked |  | 7 (11.5%) | 6 (16.7%) | 1 (4.0%) |  |
| Unchecked |  | 54 (88.5%) | 30 (83.3%) | 24 (96.0%) |  |
| Antipsychotics (choice=Olanzapine (Zyprexa)), N (%) | 0 |  |  |  |  |
| Unchecked |  | 61 (100.0%) | 36 (100.0%) | 25 (100.0%) |  |
| Antipsychotics (choice=Quetiapine (Seroquel)), N (%) | 0 |  |  |  | 0.14 |
| Checked |  | 3 (4.9%) | 3 (8.3%) | 0 (0.0%) |  |
| Unchecked |  | 58 (95.1%) | 33 (91.7%) | 25 (100.0%) |  |
| Antipsychotics (choice=Ziprasidone (Geodon)), N (%) | 0 |  |  |  |  |
| Unchecked |  | 61 (100.0%) | 36 (100.0%) | 25 (100.0%) |  |
| Antipsychotics (choice=Lurasidone (Latuda)), N (%) | 0 |  |  |  |  |
| Unchecked |  | 61 (100.0%) | 36 (100.0%) | 25 (100.0%) |  |
| Antipsychotics (choice=Paliperidone (Invega)), N (%) | 0 |  |  |  |  |
| Unchecked |  | 61 (100.0%) | 36 (100.0%) | 25 (100.0%) |  |
| Antipsychotics (choice=Asenapine (Saphris)), N (%) | 0 |  |  |  |  |
| Unchecked |  | 61 (100.0%) | 36 (100.0%) | 25 (100.0%) |  |
| Antipsychotics (choice=Iloperidone (Fanapt)), N (%) | 0 |  |  |  |  |
| Unchecked |  | 61 (100.0%) | 36 (100.0%) | 25 (100.0%) |  |
| Antipsychotics (choice=Brexpiprazole (Rexulti)), N (%) | 0 |  |  |  |  |
| Unchecked |  | 61 (100.0%) | 36 (100.0%) | 25 (100.0%) |  |
| Antipsychotics (choice=Cariprazine (Vraylar)), N (%) | 0 |  |  |  |  |
| Unchecked |  | 61 (100.0%) | 36 (100.0%) | 25 (100.0%) |  |
| Antipsychotics (choice=Haloperidol (Haldol)), N (%) | 0 |  |  |  |  |
| Unchecked |  | 61 (100.0%) | 36 (100.0%) | 25 (100.0%) |  |
| Antipsychotics (choice=Fluphenazine (Prolixin)), N (%) | 0 |  |  |  |  |
| Unchecked |  | 61 (100.0%) | 36 (100.0%) | 25 (100.0%) |  |
| Antipsychotics (choice=Chlorpromazine (Thorazine)), N (%) | 0 |  |  |  |  |
| Unchecked |  | 61 (100.0%) | 36 (100.0%) | 25 (100.0%) |  |
| Antipsychotics (choice=Thioridazine (Mellaril)), N (%) | 0 |  |  |  |  |
| Unchecked |  | 61 (100.0%) | 36 (100.0%) | 25 (100.0%) |  |
| Antipsychotics (choice=Perphenazine (Trilafon)), N (%) | 0 |  |  |  |  |
| Unchecked |  | 61 (100.0%) | 36 (100.0%) | 25 (100.0%) |  |
| Antipsychotics (choice=Molindone (Moban)), N (%) | 0 |  |  |  |  |
| Unchecked |  | 61 (100.0%) | 36 (100.0%) | 25 (100.0%) |  |
| Mood Stabilizers (choice=Lithium), N (%) | 0 |  |  |  |  |
| Unchecked |  | 61 (100.0%) | 36 (100.0%) | 25 (100.0%) |  |
| Mood Stabilizers (choice=Valproate (Depakote)), N (%) | 0 |  |  |  |  |
| Unchecked |  | 61 (100.0%) | 36 (100.0%) | 25 (100.0%) |  |
| Mood Stabilizers (choice=Lamotrigine (Lamictal)), N (%) | 0 |  |  |  |  |
| Unchecked |  | 61 (100.0%) | 36 (100.0%) | 25 (100.0%) |  |
| Mood Stabilizers (choice=Carbamazepine (Tegretol)), N (%) | 0 |  |  |  |  |
| Unchecked |  | 61 (100.0%) | 36 (100.0%) | 25 (100.0%) |  |
| Mood Stabilizers (choice=Oxcarbazepine (Trileptal)), N (%) | 0 |  |  |  |  |
| Unchecked |  | 61 (100.0%) | 36 (100.0%) | 25 (100.0%) |  |
| Mood Stabilizers (choice=Topiramate (Topamax)), N (%) | 0 |  |  |  |  |
| Unchecked |  | 61 (100.0%) | 36 (100.0%) | 25 (100.0%) |  |
| Mood Stabilizers (choice=Gabapentin (Neurontin)), N (%) | 0 |  |  |  |  |
| Unchecked |  | 61 (100.0%) | 36 (100.0%) | 25 (100.0%) |  |
| Mood Stabilizers (choice=Pregabalin (Lyrica)), N (%) | 0 |  |  |  |  |
| Unchecked |  | 61 (100.0%) | 36 (100.0%) | 25 (100.0%) |  |
| Mood Stabilizers (choice=Divalproex (Depakene)), N (%) | 0 |  |  |  |  |
| Unchecked |  | 61 (100.0%) | 36 (100.0%) | 25 (100.0%) |  |
| Anxiolytics (choice=Alprazolam (Xanax)), N (%) | 0 |  |  |  |  |
| Unchecked |  | 61 (100.0%) | 36 (100.0%) | 25 (100.0%) |  |
| Anxiolytics (choice=Clonazepam (Klonopin)), N (%) | 0 |  |  |  |  |
| Unchecked |  | 61 (100.0%) | 36 (100.0%) | 25 (100.0%) |  |
| Anxiolytics (choice=Lorazepam (Ativan)), N (%) | 0 |  |  |  |  |
| Unchecked |  | 61 (100.0%) | 36 (100.0%) | 25 (100.0%) |  |
| Anxiolytics (choice=Diazepam (Valium)), N (%) | 0 |  |  |  |  |
| Unchecked |  | 61 (100.0%) | 36 (100.0%) | 25 (100.0%) |  |
| Anxiolytics (choice=Chlordiazepoxide (Librium)), N (%) | 0 |  |  |  |  |
| Unchecked |  | 61 (100.0%) | 36 (100.0%) | 25 (100.0%) |  |
| Anxiolytics (choice=Temazepam (Restoril)), N (%) | 0 |  |  |  |  |
| Unchecked |  | 61 (100.0%) | 36 (100.0%) | 25 (100.0%) |  |
| Anxiolytics (choice=Buspirone (Buspar)), N (%) | 0 |  |  |  | 0.40 |
| Checked |  | 1 (1.6%) | 1 (2.8%) | 0 (0.0%) |  |
| Unchecked |  | 60 (98.4%) | 35 (97.2%) | 25 (100.0%) |  |
| Anxiolytics (choice=Hydroxyzine (Vistaril, Atarax)), N (%) | 0 |  |  |  |  |
| Unchecked |  | 61 (100.0%) | 36 (100.0%) | 25 (100.0%) |  |
| Talk Therapy, N (%) | 0 |  |  |  | **0.099** |
| No |  | 18 (29.5%) | 13 (36.1%) | 5 (20.0%) |  |
| Unknown |  | 34 (55.7%) | 16 (44.4%) | 18 (72.0%) |  |
| Yes |  | 9 (14.8%) | 7 (19.4%) | 2 (8.0%) |  |
| Duration of Therapy, N (%) | 60 |  |  |  |  |
| 3-6 months |  | 1 (100.0%) | 1 (100.0%) |  |  |
| Type of Birth, N (%) | 1 |  |  |  | 0.45 |
| Cesarean (c-section) |  | 15 (25.0%) | 10 (28.6%) | 5 (20.0%) |  |
| Vaginal |  | 45 (75.0%) | 25 (71.4%) | 20 (80.0%) |  |
| Reason for Cesarean Section, N (%) | 46 |  |  |  | 0.38 |
| breech presentation and cHTN w/ SI PEC w/o SF |  | 1 (6.7%) | 1 (10.0%) | 0 (0.0%) |  |
| elective |  | 1 (6.7%) | 0 (0.0%) | 1 (20.0%) |  |
| fetal bradycardia |  | 1 (6.7%) | 1 (10.0%) | 0 (0.0%) |  |
| fetal malpresentation, severe preeclampsia with severe features |  | 1 (6.7%) | 1 (10.0%) | 0 (0.0%) |  |
| fetal malpresentation, twins |  | 1 (6.7%) | 1 (10.0%) | 0 (0.0%) |  |
| hsv |  | 1 (6.7%) | 0 (0.0%) | 1 (20.0%) |  |
| hx of c-section |  | 1 (6.7%) | 1 (10.0%) | 0 (0.0%) |  |
| hx of shoulder dystocia |  | 1 (6.7%) | 0 (0.0%) | 1 (20.0%) |  |
| hysterotomy |  | 1 (6.7%) | 0 (0.0%) | 1 (20.0%) |  |
| non-assuring FHTs |  | 1 (6.7%) | 1 (10.0%) | 0 (0.0%) |  |
| non-reassuring fhts |  | 1 (6.7%) | 1 (10.0%) | 0 (0.0%) |  |
| preeclampsia |  | 1 (6.7%) | 1 (10.0%) | 0 (0.0%) |  |
| previous C-sections |  | 1 (6.7%) | 1 (10.0%) | 0 (0.0%) |  |
| repeat |  | 1 (6.7%) | 1 (10.0%) | 0 (0.0%) |  |
| terminal fetal bradycardia |  | 1 (6.7%) | 0 (0.0%) | 1 (20.0%) |  |
| NICU admission? (Gestation Age < 35 weeks), N (%) | 1 |  |  |  | 0.37 |
| No |  | 57 (95.0%) | 34 (97.1%) | 23 (92.0%) |  |
| Yes |  | 3 (5.0%) | 1 (2.9%) | 2 (8.0%) |  |
| Postpartum Hemorrhage? (< /= 1000 mL at delivery), N (%) | 1 |  |  |  | 0.13 |
| No |  | 57 (95.0%) | 32 (91.4%) | 25 (100.0%) |  |
| Yes |  | 3 (5.0%) | 3 (8.6%) | 0 (0.0%) |  |
| Severe Preeclampsia?, N (%) | 1 |  |  |  | 0.36 |
| No |  | 51 (85.0%) | 31 (88.6%) | 20 (80.0%) |  |
| Yes |  | 9 (15.0%) | 4 (11.4%) | 5 (20.0%) |  |
| Complete?, N (%) | 0 |  |  |  | 0.40 |
| Complete |  | 60 (98.4%) | 35 (97.2%) | 25 (100.0%) |  |
| Incomplete |  | 1 (1.6%) | 1 (2.8%) | 0 (0.0%) |  |
| prenatal EPDS score1, N | 15 | 46 | 30 | 16 | 0.18 |
| Mean (SD) |  | 11.7 (6.6) | 12.7 (6.2) | 9.9 (6.9) |  |
| Median (IQR) |  | 11.0 (7.0, 16.0) | 12.5 (10.0, 17.0) | 8.5 (6.0, 13.0) |  |
| Median (Range) |  | 11.0 (0.0, 25.0) | 12.5 (2.0, 25.0) | 8.5 (0.0, 23.0) |  |
| postpartum EPDS score1, N | 32 | 29 | 17 | 12 | 0.31 |
| Mean (SD) |  | 8.0 (5.6) | 7.1 (5.7) | 9.3 (5.6) |  |
| Median (IQR) |  | 7.0 (3.0, 12.0) | 6.0 (2.0, 12.0) | 9.0 (6.5, 12.0) |  |
| Median (Range) |  | 7.0 (0.0, 19.0) | 6.0 (0.0, 17.0) | 9.0 (0.0, 19.0) |  |
| lost to follow up?, N (%) | 0 |  |  |  | 0.68 |
| incomplete |  | 1 (1.6%) | 1 (2.8%) | 0 (0.0%) |  |
| no |  | 30 (49.2%) | 18 (50.0%) | 12 (48.0%) |  |
| yes |  | 30 (49.2%) | 17 (47.2%) | 13 (52.0%) |  |

|  | | | **When Referred?** | | |  |
| --- | --- | --- | --- | --- | --- | --- |
| **Variable** | **NMiss** | **Overall (n=71)** | **Before birth (n=36)** | **After birth (n=25)** | **Unknown (n=10)** | **Chisq or ANOVA p-value** |
| Have seen Dr. Tabi, N (%) | 0 |  |  |  |  | **0.0002** |
| No |  | 27 (38.0%) | 7 (19.4%) | 11 (44.0%) | 9 (90.0%) |  |
| Yes |  | 44 (62.0%) | 29 (80.6%) | 14 (56.0%) | 1 (10.0%) |  |
| When Referred?, N (%) | 10 |  |  |  |  | **<.0001** |
| After birth |  | 25 (41.0%) | 0 (0.0%) | 25 (100.0%) |  |  |
| Before birth |  | 36 (59.0%) | 36 (100.0%) | 0 (0.0%) |  |  |
| Prenatal EPDS SI, N (%) | 22 |  |  |  |  | 0.10 |
| Hardly ever |  | 9 (18.4%) | 3 (11.1%) | 2 (13.3%) | 4 (57.1%) |  |
| Never/Denies |  | 36 (73.5%) | 22 (81.5%) | 11 (73.3%) | 3 (42.9%) |  |
| Sometimes |  | 3 (6.1%) | 2 (7.4%) | 1 (6.7%) | 0 (0.0%) |  |
| Yes, quite often |  | 1 (2.0%) | 0 (0.0%) | 1 (6.7%) | 0 (0.0%) |  |
| Postpartum EPDS SI, N (%) | 40 |  |  |  |  |  |
| Never/Denies |  | 31 (100.0%) | 15 (100.0%) | 12 (100.0%) | 4 (100.0%) |  |
| Age, N | 1 | 70 | 35 | 25 | 10 | 0.88 |
| Mean (SD) |  | 27.8 (6.4) | 27.4 (6.4) | 28.1 (5.8) | 28.3 (8.4) |  |
| Median (IQR) |  | 27.5 (22.0, 33.0) | 26.0 (21.0, 32.0) | 28.0 (25.0, 32.0) | 28.0 (20.0, 35.0) |  |
| Median (Range) |  | 27.5 (17.0, 39.0) | 26.0 (18.0, 39.0) | 28.0 (17.0, 38.0) | 28.0 (20.0, 39.0) |  |
| Ethnicity (choice=Hispanic/Latino of any race), N (%) | 0 |  |  |  |  | 0.73 |
| Checked |  | 24 (33.8%) | 13 (36.1%) | 7 (28.0%) | 4 (40.0%) |  |
| Unchecked |  | 47 (66.2%) | 23 (63.9%) | 18 (72.0%) | 6 (60.0%) |  |
| Ethnicity (choice=American Indian or Alaska Native), N (%) | 0 |  |  |  |  |  |
| Unchecked |  | 71 (100.0%) | 36 (100.0%) | 25 (100.0%) | 10 (100.0%) |  |
| Ethnicity (choice=Asian), N (%) | 0 |  |  |  |  |  |
| Unchecked |  | 71 (100.0%) | 36 (100.0%) | 25 (100.0%) | 10 (100.0%) |  |
| Ethnicity (choice=Black or African American), N (%) | 0 |  |  |  |  | 0.27 |
| Checked |  | 31 (43.7%) | 17 (47.2%) | 12 (48.0%) | 2 (20.0%) |  |
| Unchecked |  | 40 (56.3%) | 19 (52.8%) | 13 (52.0%) | 8 (80.0%) |  |
| Ethnicity (choice=Native Hawaiian or Other Pacific Islander), N (%) | 0 |  |  |  |  |  |
| Unchecked |  | 71 (100.0%) | 36 (100.0%) | 25 (100.0%) | 10 (100.0%) |  |
| Ethnicity (choice=White), N (%) | 0 |  |  |  |  | 0.27 |
| Checked |  | 15 (21.1%) | 6 (16.7%) | 5 (20.0%) | 4 (40.0%) |  |
| Unchecked |  | 56 (78.9%) | 30 (83.3%) | 20 (80.0%) | 6 (60.0%) |  |
| Ethnicity (choice=Unknown), N (%) | 0 |  |  |  |  | 0.81 |
| Checked |  | 2 (2.8%) | 1 (2.8%) | 1 (4.0%) | 0 (0.0%) |  |
| Unchecked |  | 69 (97.2%) | 35 (97.2%) | 24 (96.0%) | 10 (100.0%) |  |
| Employed?, N (%) | 0 |  |  |  |  | 0.10 |
| No |  | 42 (59.2%) | 22 (61.1%) | 11 (44.0%) | 9 (90.0%) |  |
| Unknown |  | 9 (12.7%) | 3 (8.3%) | 5 (20.0%) | 1 (10.0%) |  |
| Yes |  | 20 (28.2%) | 11 (30.6%) | 9 (36.0%) | 0 (0.0%) |  |
| Unhoused?, N (%) | 0 |  |  |  |  | 0.40 |
| No |  | 66 (93.0%) | 34 (94.4%) | 22 (88.0%) | 10 (100.0%) |  |
| Unknown |  | 5 (7.0%) | 2 (5.6%) | 3 (12.0%) | 0 (0.0%) |  |
| Substance use?, N (%) | 0 |  |  |  |  | **0.024** |
| No |  | 20 (28.2%) | 11 (30.6%) | 8 (32.0%) | 1 (10.0%) |  |
| Unknown |  | 17 (23.9%) | 4 (11.1%) | 7 (28.0%) | 6 (60.0%) |  |
| Yes |  | 34 (47.9%) | 21 (58.3%) | 10 (40.0%) | 3 (30.0%) |  |
| Substance use during pregnancy?, N (%) | 37 |  |  |  |  | **0.094** |
| No |  | 12 (35.3%) | 6 (28.6%) | 6 (60.0%) | 0 (0.0%) |  |
| Yes |  | 22 (64.7%) | 15 (71.4%) | 4 (40.0%) | 3 (100.0%) |  |
| Substance Type? (choice=Alcohol), N (%) | 0 |  |  |  |  | 0.31 |
| Checked |  | 10 (14.1%) | 5 (13.9%) | 5 (20.0%) | 0 (0.0%) |  |
| Unchecked |  | 61 (85.9%) | 31 (86.1%) | 20 (80.0%) | 10 (100.0%) |  |
| Substance Type? (choice=Caffeine), N (%) | 0 |  |  |  |  | 0.61 |
| Checked |  | 1 (1.4%) | 1 (2.8%) | 0 (0.0%) | 0 (0.0%) |  |
| Unchecked |  | 70 (98.6%) | 35 (97.2%) | 25 (100.0%) | 10 (100.0%) |  |
| Substance Type? (choice=Cannabis (Marijuana)), N (%) | 0 |  |  |  |  | **0.030** |
| Checked |  | 25 (35.2%) | 18 (50.0%) | 5 (20.0%) | 2 (20.0%) |  |
| Unchecked |  | 46 (64.8%) | 18 (50.0%) | 20 (80.0%) | 8 (80.0%) |  |
| Substance Type? (choice=LSD (Lysergic Acid Diethylamide)), N (%) | 0 |  |  |  |  |  |
| Unchecked |  | 71 (100.0%) | 36 (100.0%) | 25 (100.0%) | 10 (100.0%) |  |
| Substance Type? (choice=Psilocybin (Magic Mushrooms)), N (%) | 0 |  |  |  |  |  |
| Unchecked |  | 71 (100.0%) | 36 (100.0%) | 25 (100.0%) | 10 (100.0%) |  |
| Substance Type? (choice=Phencyclidine (PCP)), N (%) | 0 |  |  |  |  | 0.61 |
| Checked |  | 1 (1.4%) | 1 (2.8%) | 0 (0.0%) | 0 (0.0%) |  |
| Unchecked |  | 70 (98.6%) | 35 (97.2%) | 25 (100.0%) | 10 (100.0%) |  |
| Substance Type? (choice=Ketamine), N (%) | 0 |  |  |  |  |  |
| Unchecked |  | 71 (100.0%) | 36 (100.0%) | 25 (100.0%) | 10 (100.0%) |  |
| Substance Type? (choice=Inhalants (e.g., paint thinner, gasoline fumes)), N (%) | 0 |  |  |  |  |  |
| Unchecked |  | 71 (100.0%) | 36 (100.0%) | 25 (100.0%) | 10 (100.0%) |  |
| Substance Type? (choice=Opioids (e.g., heroin, prescription pain medications)), N (%) | 0 |  |  |  |  | 0.23 |
| Checked |  | 6 (8.5%) | 5 (13.9%) | 1 (4.0%) | 0 (0.0%) |  |
| Unchecked |  | 65 (91.5%) | 31 (86.1%) | 24 (96.0%) | 10 (100.0%) |  |
| Substance Type? (choice=Benzodiazepines (e.g., Xanax, Valium)), N (%) | 0 |  |  |  |  | 0.65 |
| Checked |  | 4 (5.6%) | 2 (5.6%) | 2 (8.0%) | 0 (0.0%) |  |
| Unchecked |  | 67 (94.4%) | 34 (94.4%) | 23 (92.0%) | 10 (100.0%) |  |
| Substance Type? (choice=Barbiturates (e.g., Phenobarbital)), N (%) | 0 |  |  |  |  |  |
| Unchecked |  | 71 (100.0%) | 36 (100.0%) | 25 (100.0%) | 10 (100.0%) |  |
| Substance Type? (choice=Non-Benzodiazepine Hypnotics (e.g., Ambien, Lunesta)), N (%) | 0 |  |  |  |  |  |
| Unchecked |  | 71 (100.0%) | 36 (100.0%) | 25 (100.0%) | 10 (100.0%) |  |
| Substance Type? (choice=Amphetamines (e.g., Adderall, methamphetamine)), N (%) | 0 |  |  |  |  | 0.37 |
| Checked |  | 2 (2.8%) | 2 (5.6%) | 0 (0.0%) | 0 (0.0%) |  |
| Unchecked |  | 69 (97.2%) | 34 (94.4%) | 25 (100.0%) | 10 (100.0%) |  |
| Substance Type? (choice=Cocaine), N (%) | 0 |  |  |  |  | 0.51 |
| Checked |  | 6 (8.5%) | 3 (8.3%) | 3 (12.0%) | 0 (0.0%) |  |
| Unchecked |  | 65 (91.5%) | 33 (91.7%) | 22 (88.0%) | 10 (100.0%) |  |
| Substance Type? (choice=Tobacco (Nicotine)), N (%) | 0 |  |  |  |  | 0.58 |
| Checked |  | 9 (12.7%) | 6 (16.7%) | 2 (8.0%) | 1 (10.0%) |  |
| Unchecked |  | 62 (87.3%) | 30 (83.3%) | 23 (92.0%) | 9 (90.0%) |  |
| Substance Type? (choice=Synthetic Cannabinoids (e.g., Spice, K2)), N (%) | 0 |  |  |  |  |  |
| Unchecked |  | 71 (100.0%) | 36 (100.0%) | 25 (100.0%) | 10 (100.0%) |  |
| Substance Type? (choice=Synthetic Cathinones (e.g., Bath Salts)), N (%) | 0 |  |  |  |  |  |
| Unchecked |  | 71 (100.0%) | 36 (100.0%) | 25 (100.0%) | 10 (100.0%) |  |
| Substance Type? (choice=Other or Unknown Substances), N (%) | 0 |  |  |  |  |  |
| Unchecked |  | 71 (100.0%) | 36 (100.0%) | 25 (100.0%) | 10 (100.0%) |  |
| Method of Substance Use? (choice=Inhalation (e.g., smoking, vaping)), N (%) | 0 |  |  |  |  | 0.61 |
| Checked |  | 1 (1.4%) | 1 (2.8%) | 0 (0.0%) | 0 (0.0%) |  |
| Unchecked |  | 70 (98.6%) | 35 (97.2%) | 25 (100.0%) | 10 (100.0%) |  |
| Method of Substance Use? (choice=Oral Ingestion (e.g., swallowing pills or liquids)), N (%) | 0 |  |  |  |  |  |
| Unchecked |  | 71 (100.0%) | 36 (100.0%) | 25 (100.0%) | 10 (100.0%) |  |
| Method of Substance Use? (choice=Intranasal (e.g., snorting or sniffing)), N (%) | 0 |  |  |  |  |  |
| Unchecked |  | 71 (100.0%) | 36 (100.0%) | 25 (100.0%) | 10 (100.0%) |  |
| Method of Substance Use? (choice=Intravenous (IV) Injection), N (%) | 0 |  |  |  |  |  |
| Unchecked |  | 71 (100.0%) | 36 (100.0%) | 25 (100.0%) | 10 (100.0%) |  |
| Method of Substance Use? (choice=Intramuscular (IM) Injection), N (%) | 0 |  |  |  |  |  |
| Unchecked |  | 71 (100.0%) | 36 (100.0%) | 25 (100.0%) | 10 (100.0%) |  |
| Method of Substance Use? (choice=Subcutaneous (SC) Injection), N (%) | 0 |  |  |  |  |  |
| Unchecked |  | 71 (100.0%) | 36 (100.0%) | 25 (100.0%) | 10 (100.0%) |  |
| Method of Substance Use? (choice=Transdermal (e.g., patches)), N (%) | 0 |  |  |  |  |  |
| Unchecked |  | 71 (100.0%) | 36 (100.0%) | 25 (100.0%) | 10 (100.0%) |  |
| Method of Substance Use? (choice=Rectal (e.g., suppositories)), N (%) | 0 |  |  |  |  |  |
| Unchecked |  | 71 (100.0%) | 36 (100.0%) | 25 (100.0%) | 10 (100.0%) |  |
| Method of Substance Use? (choice=Sublingual (under the tongue)), N (%) | 0 |  |  |  |  |  |
| Unchecked |  | 71 (100.0%) | 36 (100.0%) | 25 (100.0%) | 10 (100.0%) |  |
| Method of Substance Use? (choice=Transbuccal (between the cheek and gum)), N (%) | 0 |  |  |  |  |  |
| Unchecked |  | 71 (100.0%) | 36 (100.0%) | 25 (100.0%) | 10 (100.0%) |  |
| History of abuse?, N (%) | 0 |  |  |  |  | 0.11 |
| No |  | 17 (23.9%) | 9 (25.0%) | 6 (24.0%) | 2 (20.0%) |  |
| Unknown |  | 31 (43.7%) | 13 (36.1%) | 10 (40.0%) | 8 (80.0%) |  |
| Yes |  | 23 (32.4%) | 14 (38.9%) | 9 (36.0%) | 0 (0.0%) |  |
| Relationship Status, N (%) | 0 |  |  |  |  | **0.043** |
| Divorced |  | 1 (1.4%) | 0 (0.0%) | 1 (4.0%) | 0 (0.0%) |  |
| Has long-term partner |  | 33 (46.5%) | 20 (55.6%) | 12 (48.0%) | 1 (10.0%) |  |
| Married |  | 5 (7.0%) | 3 (8.3%) | 2 (8.0%) | 0 (0.0%) |  |
| Single |  | 9 (12.7%) | 6 (16.7%) | 2 (8.0%) | 1 (10.0%) |  |
| Unknown |  | 23 (32.4%) | 7 (19.4%) | 8 (32.0%) | 8 (80.0%) |  |
| Education Level, N (%) | 0 |  |  |  |  | 0.21 |
| Associates degree |  | 2 (2.8%) | 1 (2.8%) | 1 (4.0%) | 0 (0.0%) |  |
| Bachelors degree |  | 1 (1.4%) | 0 (0.0%) | 1 (4.0%) | 0 (0.0%) |  |
| Below High School |  | 10 (14.1%) | 7 (19.4%) | 2 (8.0%) | 1 (10.0%) |  |
| Graduate Degree |  | 2 (2.8%) | 0 (0.0%) | 2 (8.0%) | 0 (0.0%) |  |
| High School |  | 16 (22.5%) | 11 (30.6%) | 5 (20.0%) | 0 (0.0%) |  |
| Unknown |  | 40 (56.3%) | 17 (47.2%) | 14 (56.0%) | 9 (90.0%) |  |
| Previous IP hospitalizations?, N (%) | 1 |  |  |  |  | 0.11 |
| Found |  | 12 (17.1%) | 9 (25.7%) | 3 (12.0%) | 0 (0.0%) |  |
| Not Found |  | 58 (82.9%) | 26 (74.3%) | 22 (88.0%) | 10 (100.0%) |  |
| Psychiatric Diagnosis (choice=None), N (%) | 0 |  |  |  |  | **0.001** |
| Checked |  | 22 (31.0%) | 7 (19.4%) | 7 (28.0%) | 8 (80.0%) |  |
| Unchecked |  | 49 (69.0%) | 29 (80.6%) | 18 (72.0%) | 2 (20.0%) |  |
| Psychiatric Diagnosis (choice=Bipolar Disorders), N (%) | 0 |  |  |  |  | **0.007** |
| Checked |  | 9 (12.7%) | 9 (25.0%) | 0 (0.0%) | 0 (0.0%) |  |
| Unchecked |  | 62 (87.3%) | 27 (75.0%) | 25 (100.0%) | 10 (100.0%) |  |
| Psychiatric Diagnosis (choice=Major Depressive Disorder (MDD)), N (%) | 0 |  |  |  |  | 0.10 |
| Checked |  | 28 (39.4%) | 17 (47.2%) | 10 (40.0%) | 1 (10.0%) |  |
| Unchecked |  | 43 (60.6%) | 19 (52.8%) | 15 (60.0%) | 9 (90.0%) |  |
| Psychiatric Diagnosis (choice=Schizophrenia and Other Psychotic Disorders), N (%) | 0 |  |  |  |  | **0.073** |
| Checked |  | 5 (7.0%) | 5 (13.9%) | 0 (0.0%) | 0 (0.0%) |  |
| Unchecked |  | 66 (93.0%) | 31 (86.1%) | 25 (100.0%) | 10 (100.0%) |  |
| Psychiatric Diagnosis (choice=Generalized Anxiety Disorder (GAD)), N (%) | 0 |  |  |  |  | 0.20 |
| Checked |  | 25 (35.2%) | 14 (38.9%) | 10 (40.0%) | 1 (10.0%) |  |
| Unchecked |  | 46 (64.8%) | 22 (61.1%) | 15 (60.0%) | 9 (90.0%) |  |
| Psychiatric Diagnosis (choice=Borderline Personality Disorder), N (%) | 0 |  |  |  |  | 0.39 |
| Checked |  | 1 (1.4%) | 0 (0.0%) | 1 (4.0%) | 0 (0.0%) |  |
| Unchecked |  | 70 (98.6%) | 36 (100.0%) | 24 (96.0%) | 10 (100.0%) |  |
| Psychiatric Diagnosis (choice=Substance Use Disorder), N (%) | 0 |  |  |  |  | 0.40 |
| Checked |  | 8 (11.3%) | 4 (11.1%) | 4 (16.0%) | 0 (0.0%) |  |
| Unchecked |  | 63 (88.7%) | 32 (88.9%) | 21 (84.0%) | 10 (100.0%) |  |
| Psychiatric Diagnosis (choice=Obsessive Compulsive Disorder (OCD)), N (%) | 0 |  |  |  |  |  |
| Unchecked |  | 71 (100.0%) | 36 (100.0%) | 25 (100.0%) | 10 (100.0%) |  |
| Psychiatric Diagnosis (choice=Posttraumatic Stress Disorder (PTSD)), N (%) | 0 |  |  |  |  | **0.080** |
| Checked |  | 20 (28.2%) | 13 (36.1%) | 7 (28.0%) | 0 (0.0%) |  |
| Unchecked |  | 51 (71.8%) | 23 (63.9%) | 18 (72.0%) | 10 (100.0%) |  |
| Psychiatric Diagnosis (choice=postpartum depressive disorder), N (%) | 0 |  |  |  |  | **0.013** |
| Checked |  | 7 (9.9%) | 1 (2.8%) | 6 (24.0%) | 0 (0.0%) |  |
| Unchecked |  | 64 (90.1%) | 35 (97.2%) | 19 (76.0%) | 10 (100.0%) |  |
| Diagnoser, N (%) | 21 |  |  |  |  | **0.035** |
| Dr. Tabi |  | 33 (66.0%) | 22 (73.3%) | 11 (61.1%) | 0 (0.0%) |  |
| Other |  | 9 (18.0%) | 4 (13.3%) | 3 (16.7%) | 2 (100.0%) |  |
| Self-reported |  | 8 (16.0%) | 4 (13.3%) | 4 (22.2%) | 0 (0.0%) |  |
| Bipolar Disorders (choice=Bipolar I Disorder), N (%) | 0 |  |  |  |  | 0.37 |
| Checked |  | 2 (2.8%) | 2 (5.6%) | 0 (0.0%) | 0 (0.0%) |  |
| Unchecked |  | 69 (97.2%) | 34 (94.4%) | 25 (100.0%) | 10 (100.0%) |  |
| Bipolar Disorders (choice=Bipolar II Disorder), N (%) | 0 |  |  |  |  | 0.61 |
| Checked |  | 1 (1.4%) | 1 (2.8%) | 0 (0.0%) | 0 (0.0%) |  |
| Unchecked |  | 70 (98.6%) | 35 (97.2%) | 25 (100.0%) | 10 (100.0%) |  |
| Bipolar Disorders (choice=Cyclothymic Disorder (Cyclothymia)), N (%) | 0 |  |  |  |  |  |
| Unchecked |  | 71 (100.0%) | 36 (100.0%) | 25 (100.0%) | 10 (100.0%) |  |
| Bipolar Disorders (choice=Other Specified Bipolar and Related Disorder), N (%) | 0 |  |  |  |  | 0.61 |
| Checked |  | 1 (1.4%) | 1 (2.8%) | 0 (0.0%) | 0 (0.0%) |  |
| Unchecked |  | 70 (98.6%) | 35 (97.2%) | 25 (100.0%) | 10 (100.0%) |  |
| Bipolar Disorders (choice=Unspecified Bipolar and Related Disorder), N (%) | 0 |  |  |  |  | 0.37 |
| Checked |  | 2 (2.8%) | 2 (5.6%) | 0 (0.0%) | 0 (0.0%) |  |
| Unchecked |  | 69 (97.2%) | 34 (94.4%) | 25 (100.0%) | 10 (100.0%) |  |
| Substance Use Disorders (per DSM5) (choice=Alcohol Use Disorder), N (%) | 0 |  |  |  |  | 0.39 |
| Checked |  | 1 (1.4%) | 0 (0.0%) | 1 (4.0%) | 0 (0.0%) |  |
| Unchecked |  | 70 (98.6%) | 36 (100.0%) | 24 (96.0%) | 10 (100.0%) |  |
| Substance Use Disorders (per DSM5) (choice=Caffeine Use Disorder), N (%) | 0 |  |  |  |  |  |
| Unchecked |  | 71 (100.0%) | 36 (100.0%) | 25 (100.0%) | 10 (100.0%) |  |
| Substance Use Disorders (per DSM5) (choice=Cannabis Use Disorder), N (%) | 0 |  |  |  |  | 0.74 |
| Checked |  | 3 (4.2%) | 2 (5.6%) | 1 (4.0%) | 0 (0.0%) |  |
| Unchecked |  | 68 (95.8%) | 34 (94.4%) | 24 (96.0%) | 10 (100.0%) |  |
| Substance Use Disorders (per DSM5) (choice=Hallucinogen Use Disorder), N (%) | 0 |  |  |  |  | 0.61 |
| Checked |  | 1 (1.4%) | 1 (2.8%) | 0 (0.0%) | 0 (0.0%) |  |
| Unchecked |  | 70 (98.6%) | 35 (97.2%) | 25 (100.0%) | 10 (100.0%) |  |
| Substance Use Disorders (per DSM5) (choice=Inhalant Use Disorder), N (%) | 0 |  |  |  |  |  |
| Unchecked |  | 71 (100.0%) | 36 (100.0%) | 25 (100.0%) | 10 (100.0%) |  |
| Substance Use Disorders (per DSM5) (choice=Opioid Use Disorder), N (%) | 0 |  |  |  |  | 0.81 |
| Checked |  | 2 (2.8%) | 1 (2.8%) | 1 (4.0%) | 0 (0.0%) |  |
| Unchecked |  | 69 (97.2%) | 35 (97.2%) | 24 (96.0%) | 10 (100.0%) |  |
| Substance Use Disorders (per DSM5) (choice=Sedative, Hypnotic, or Anxiolytic Use Disorder), N (%) | 0 |  |  |  |  | 0.39 |
| Checked |  | 1 (1.4%) | 0 (0.0%) | 1 (4.0%) | 0 (0.0%) |  |
| Unchecked |  | 70 (98.6%) | 36 (100.0%) | 24 (96.0%) | 10 (100.0%) |  |
| Substance Use Disorders (per DSM5) (choice=Stimulant Use Disorder), N (%) | 0 |  |  |  |  | 0.81 |
| Checked |  | 2 (2.8%) | 1 (2.8%) | 1 (4.0%) | 0 (0.0%) |  |
| Unchecked |  | 69 (97.2%) | 35 (97.2%) | 24 (96.0%) | 10 (100.0%) |  |
| Substance Use Disorders (per DSM5) (choice=Tobacco Use Disorder), N (%) | 0 |  |  |  |  | 0.74 |
| Checked |  | 3 (4.2%) | 2 (5.6%) | 1 (4.0%) | 0 (0.0%) |  |
| Unchecked |  | 68 (95.8%) | 34 (94.4%) | 24 (96.0%) | 10 (100.0%) |  |
| Substance Use Disorders (per DSM5) (choice=Other (or Unknown) Substance Use Disorder), N (%) | 0 |  |  |  |  |  |
| Unchecked |  | 71 (100.0%) | 36 (100.0%) | 25 (100.0%) | 10 (100.0%) |  |
| Substance Use Disorders (per DSM5) (choice=Alcohol Intoxication), N (%) | 0 |  |  |  |  |  |
| Unchecked |  | 71 (100.0%) | 36 (100.0%) | 25 (100.0%) | 10 (100.0%) |  |
| Substance Use Disorders (per DSM5) (choice=Caffeine Intoxication), N (%) | 0 |  |  |  |  |  |
| Unchecked |  | 71 (100.0%) | 36 (100.0%) | 25 (100.0%) | 10 (100.0%) |  |
| Substance Use Disorders (per DSM5) (choice=Cannabis Intoxication), N (%) | 0 |  |  |  |  |  |
| Unchecked |  | 71 (100.0%) | 36 (100.0%) | 25 (100.0%) | 10 (100.0%) |  |
| Substance Use Disorders (per DSM5) (choice=Stimulant Intoxication (e.g., amphetamines, cocaine)), N (%) | 0 |  |  |  |  |  |
| Unchecked |  | 71 (100.0%) | 36 (100.0%) | 25 (100.0%) | 10 (100.0%) |  |
| Substance Use Disorders (per DSM5) (choice=Opioid Intoxication), N (%) | 0 |  |  |  |  |  |
| Unchecked |  | 71 (100.0%) | 36 (100.0%) | 25 (100.0%) | 10 (100.0%) |  |
| Substance Use Disorders (per DSM5) (choice=Sedative, Hypnotic, or Anxiolytic Intoxication), N (%) | 0 |  |  |  |  |  |
| Unchecked |  | 71 (100.0%) | 36 (100.0%) | 25 (100.0%) | 10 (100.0%) |  |
| Substance Use Disorders (per DSM5) (choice=Hallucinogen Intoxication), N (%) | 0 |  |  |  |  |  |
| Unchecked |  | 71 (100.0%) | 36 (100.0%) | 25 (100.0%) | 10 (100.0%) |  |
| Substance Use Disorders (per DSM5) (choice=Inhalant Intoxication), N (%) | 0 |  |  |  |  |  |
| Unchecked |  | 71 (100.0%) | 36 (100.0%) | 25 (100.0%) | 10 (100.0%) |  |
| Substance Use Disorders (per DSM5) (choice=Caffeine Intoxication), N (%) | 0 |  |  |  |  |  |
| Unchecked |  | 71 (100.0%) | 36 (100.0%) | 25 (100.0%) | 10 (100.0%) |  |
| Substance Use Disorders (per DSM5) (choice=Other (or Unknown) Substance Intoxication), N (%) | 0 |  |  |  |  |  |
| Unchecked |  | 71 (100.0%) | 36 (100.0%) | 25 (100.0%) | 10 (100.0%) |  |
| Substance Use Disorders (per DSM5) (choice=Alcohol Withdrawal), N (%) | 0 |  |  |  |  |  |
| Unchecked |  | 71 (100.0%) | 36 (100.0%) | 25 (100.0%) | 10 (100.0%) |  |
| Substance Use Disorders (per DSM5) (choice=Caffeine Withdrawal), N (%) | 0 |  |  |  |  |  |
| Unchecked |  | 71 (100.0%) | 36 (100.0%) | 25 (100.0%) | 10 (100.0%) |  |
| Substance Use Disorders (per DSM5) (choice=Cannabis Withdrawal), N (%) | 0 |  |  |  |  |  |
| Unchecked |  | 71 (100.0%) | 36 (100.0%) | 25 (100.0%) | 10 (100.0%) |  |
| Substance Use Disorders (per DSM5) (choice=Stimulant Withdrawal (e.g., amphetamines, cocaine)), N (%) | 0 |  |  |  |  |  |
| Unchecked |  | 71 (100.0%) | 36 (100.0%) | 25 (100.0%) | 10 (100.0%) |  |
| Substance Use Disorders (per DSM5) (choice=Opioid Withdrawal), N (%) | 0 |  |  |  |  |  |
| Unchecked |  | 71 (100.0%) | 36 (100.0%) | 25 (100.0%) | 10 (100.0%) |  |
| Substance Use Disorders (per DSM5) (choice=Sedative, Hypnotic, or Anxiolytic Withdrawal), N (%) | 0 |  |  |  |  |  |
| Unchecked |  | 71 (100.0%) | 36 (100.0%) | 25 (100.0%) | 10 (100.0%) |  |
| Substance Use Disorders (per DSM5) (choice=Hallucinogen Persisting Perception Disorder (not strictly withdrawal but related)), N (%) | 0 |  |  |  |  |  |
| Unchecked |  | 71 (100.0%) | 36 (100.0%) | 25 (100.0%) | 10 (100.0%) |  |
| Substance Use Disorders (per DSM5) (choice=Inhalant Withdrawal), N (%) | 0 |  |  |  |  |  |
| Unchecked |  | 71 (100.0%) | 36 (100.0%) | 25 (100.0%) | 10 (100.0%) |  |
| Substance Use Disorders (per DSM5) (choice=Caffeine Withdrawal), N (%) | 0 |  |  |  |  |  |
| Unchecked |  | 71 (100.0%) | 36 (100.0%) | 25 (100.0%) | 10 (100.0%) |  |
| Substance Use Disorders (per DSM5) (choice=Other (or Unknown) Substance Withdrawal), N (%) | 0 |  |  |  |  |  |
| Unchecked |  | 71 (100.0%) | 36 (100.0%) | 25 (100.0%) | 10 (100.0%) |  |
| Psychotropic Medication (choice=None), N (%) | 0 |  |  |  |  | **0.017** |
| Checked |  | 36 (50.7%) | 14 (38.9%) | 13 (52.0%) | 9 (90.0%) |  |
| Unchecked |  | 35 (49.3%) | 22 (61.1%) | 12 (48.0%) | 1 (10.0%) |  |
| Psychotropic Medication (choice=Antidepressant (e.g., SSRI, SNRI, TCA, MAOIs, etc)), N (%) | 0 |  |  |  |  | 0.29 |
| Checked |  | 22 (31.0%) | 12 (33.3%) | 9 (36.0%) | 1 (10.0%) |  |
| Unchecked |  | 49 (69.0%) | 24 (66.7%) | 16 (64.0%) | 9 (90.0%) |  |
| Psychotropic Medication (choice=Antipsychotic (e.g., FGA, SGA)), N (%) | 0 |  |  |  |  | **0.079** |
| Checked |  | 13 (18.3%) | 10 (27.8%) | 3 (12.0%) | 0 (0.0%) |  |
| Unchecked |  | 58 (81.7%) | 26 (72.2%) | 22 (88.0%) | 10 (100.0%) |  |
| Psychotropic Medication (choice=Mood Stabilizer (e.g., Lithium, AEDs)), N (%) | 0 |  |  |  |  |  |
| Unchecked |  | 71 (100.0%) | 36 (100.0%) | 25 (100.0%) | 10 (100.0%) |  |
| Psychotropic Medication (choice=Anxiolytic (e.g., Benzos, Atarax, Buspirone)), N (%) | 0 |  |  |  |  | 0.81 |
| Checked |  | 2 (2.8%) | 1 (2.8%) | 1 (4.0%) | 0 (0.0%) |  |
| Unchecked |  | 69 (97.2%) | 35 (97.2%) | 24 (96.0%) | 10 (100.0%) |  |
| Psychotropic Medication (choice=Sedative (e.g., Ambien)), N (%) | 0 |  |  |  |  | 0.39 |
| Checked |  | 1 (1.4%) | 0 (0.0%) | 1 (4.0%) | 0 (0.0%) |  |
| Unchecked |  | 70 (98.6%) | 36 (100.0%) | 24 (96.0%) | 10 (100.0%) |  |
| Psychotropic Medication (choice=Stimulant), N (%) | 0 |  |  |  |  | 0.81 |
| Checked |  | 2 (2.8%) | 1 (2.8%) | 1 (4.0%) | 0 (0.0%) |  |
| Unchecked |  | 69 (97.2%) | 35 (97.2%) | 24 (96.0%) | 10 (100.0%) |  |
| Antidepressants (choice=Fluoxetine (Prozac)), N (%) | 0 |  |  |  |  | 0.74 |
| Checked |  | 3 (4.2%) | 2 (5.6%) | 1 (4.0%) | 0 (0.0%) |  |
| Unchecked |  | 68 (95.8%) | 34 (94.4%) | 24 (96.0%) | 10 (100.0%) |  |
| Antidepressants (choice=Sertraline (Zoloft)), N (%) | 0 |  |  |  |  | 0.32 |
| Checked |  | 11 (15.5%) | 7 (19.4%) | 4 (16.0%) | 0 (0.0%) |  |
| Unchecked |  | 60 (84.5%) | 29 (80.6%) | 21 (84.0%) | 10 (100.0%) |  |
| Antidepressants (choice=Paroxetine (Paxil)), N (%) | 0 |  |  |  |  |  |
| Unchecked |  | 71 (100.0%) | 36 (100.0%) | 25 (100.0%) | 10 (100.0%) |  |
| Antidepressants (choice=Escitalopram (Lexapro)), N (%) | 0 |  |  |  |  | 0.56 |
| Checked |  | 4 (5.6%) | 1 (2.8%) | 2 (8.0%) | 1 (10.0%) |  |
| Unchecked |  | 67 (94.4%) | 35 (97.2%) | 23 (92.0%) | 9 (90.0%) |  |
| Antidepressants (choice=Citalopram (Celexa)), N (%) | 0 |  |  |  |  |  |
| Unchecked |  | 71 (100.0%) | 36 (100.0%) | 25 (100.0%) | 10 (100.0%) |  |
| Antidepressants (choice=Fluvoxamine (Luvox)), N (%) | 0 |  |  |  |  |  |
| Unchecked |  | 71 (100.0%) | 36 (100.0%) | 25 (100.0%) | 10 (100.0%) |  |
| Antidepressants (choice=Venlafaxine (Effexor)), N (%) | 0 |  |  |  |  | 0.39 |
| Checked |  | 1 (1.4%) | 0 (0.0%) | 1 (4.0%) | 0 (0.0%) |  |
| Unchecked |  | 70 (98.6%) | 36 (100.0%) | 24 (96.0%) | 10 (100.0%) |  |
| Antidepressants (choice=Duloxetine (Cymbalta)), N (%) | 0 |  |  |  |  |  |
| Unchecked |  | 71 (100.0%) | 36 (100.0%) | 25 (100.0%) | 10 (100.0%) |  |
| Antidepressants (choice=Desvenlafaxine (Pristiq)), N (%) | 0 |  |  |  |  |  |
| Unchecked |  | 71 (100.0%) | 36 (100.0%) | 25 (100.0%) | 10 (100.0%) |  |
| Antidepressants (choice=Levomilnacipran (Fetzima)), N (%) | 0 |  |  |  |  |  |
| Unchecked |  | 71 (100.0%) | 36 (100.0%) | 25 (100.0%) | 10 (100.0%) |  |
| Antidepressants (choice=Amitriptyline (Elavil)), N (%) | 0 |  |  |  |  |  |
| Unchecked |  | 71 (100.0%) | 36 (100.0%) | 25 (100.0%) | 10 (100.0%) |  |
| Antidepressants (choice=Imipramine (Tofranil)), N (%) | 0 |  |  |  |  |  |
| Unchecked |  | 71 (100.0%) | 36 (100.0%) | 25 (100.0%) | 10 (100.0%) |  |
| Antidepressants (choice=Nortriptyline (Pamelor)), N (%) | 0 |  |  |  |  |  |
| Unchecked |  | 71 (100.0%) | 36 (100.0%) | 25 (100.0%) | 10 (100.0%) |  |
| Antidepressants (choice=Clomipramine (Anafranil)), N (%) | 0 |  |  |  |  |  |
| Unchecked |  | 71 (100.0%) | 36 (100.0%) | 25 (100.0%) | 10 (100.0%) |  |
| Antidepressants (choice=Doxepin (Sinequan)), N (%) | 0 |  |  |  |  |  |
| Unchecked |  | 71 (100.0%) | 36 (100.0%) | 25 (100.0%) | 10 (100.0%) |  |
| Antidepressants (choice=Phenelzine (Nardil)), N (%) | 0 |  |  |  |  |  |
| Unchecked |  | 71 (100.0%) | 36 (100.0%) | 25 (100.0%) | 10 (100.0%) |  |
| Antidepressants (choice=Tranylcypromine (Parnate)), N (%) | 0 |  |  |  |  |  |
| Unchecked |  | 71 (100.0%) | 36 (100.0%) | 25 (100.0%) | 10 (100.0%) |  |
| Antidepressants (choice=Isocarboxazid (Marplan)), N (%) | 0 |  |  |  |  |  |
| Unchecked |  | 71 (100.0%) | 36 (100.0%) | 25 (100.0%) | 10 (100.0%) |  |
| Antidepressants (choice=Selegiline (Emsam)), N (%) | 0 |  |  |  |  |  |
| Unchecked |  | 71 (100.0%) | 36 (100.0%) | 25 (100.0%) | 10 (100.0%) |  |
| Antidepressants (choice=Bupropion (Wellbutrin)), N (%) | 0 |  |  |  |  | 0.74 |
| Checked |  | 3 (4.2%) | 2 (5.6%) | 1 (4.0%) | 0 (0.0%) |  |
| Unchecked |  | 68 (95.8%) | 34 (94.4%) | 24 (96.0%) | 10 (100.0%) |  |
| Antidepressants (choice=Mirtazapine (Remeron)), N (%) | 0 |  |  |  |  |  |
| Unchecked |  | 71 (100.0%) | 36 (100.0%) | 25 (100.0%) | 10 (100.0%) |  |
| Antidepressants (choice=Trazodone (Desyrel)), N (%) | 0 |  |  |  |  |  |
| Unchecked |  | 71 (100.0%) | 36 (100.0%) | 25 (100.0%) | 10 (100.0%) |  |
| Antidepressants (choice=Vilazodone (Viibryd)), N (%) | 0 |  |  |  |  |  |
| Unchecked |  | 71 (100.0%) | 36 (100.0%) | 25 (100.0%) | 10 (100.0%) |  |
| Antidepressants (choice=Vortioxetine (Trintellix)), N (%) | 0 |  |  |  |  |  |
| Unchecked |  | 71 (100.0%) | 36 (100.0%) | 25 (100.0%) | 10 (100.0%) |  |
| Antipsychotics (choice=Aripiprazole (Abilify)), N (%) | 0 |  |  |  |  | 0.47 |
| Checked |  | 3 (4.2%) | 1 (2.8%) | 2 (8.0%) | 0 (0.0%) |  |
| Unchecked |  | 68 (95.8%) | 35 (97.2%) | 23 (92.0%) | 10 (100.0%) |  |
| Antipsychotics (choice=Risperidone (Risperdal)), N (%) | 0 |  |  |  |  | 0.14 |
| Checked |  | 7 (9.9%) | 6 (16.7%) | 1 (4.0%) | 0 (0.0%) |  |
| Unchecked |  | 64 (90.1%) | 30 (83.3%) | 24 (96.0%) | 10 (100.0%) |  |
| Antipsychotics (choice=Olanzapine (Zyprexa)), N (%) | 0 |  |  |  |  |  |
| Unchecked |  | 71 (100.0%) | 36 (100.0%) | 25 (100.0%) | 10 (100.0%) |  |
| Antipsychotics (choice=Quetiapine (Seroquel)), N (%) | 0 |  |  |  |  | 0.22 |
| Checked |  | 3 (4.2%) | 3 (8.3%) | 0 (0.0%) | 0 (0.0%) |  |
| Unchecked |  | 68 (95.8%) | 33 (91.7%) | 25 (100.0%) | 10 (100.0%) |  |
| Antipsychotics (choice=Ziprasidone (Geodon)), N (%) | 0 |  |  |  |  |  |
| Unchecked |  | 71 (100.0%) | 36 (100.0%) | 25 (100.0%) | 10 (100.0%) |  |
| Antipsychotics (choice=Lurasidone (Latuda)), N (%) | 0 |  |  |  |  |  |
| Unchecked |  | 71 (100.0%) | 36 (100.0%) | 25 (100.0%) | 10 (100.0%) |  |
| Antipsychotics (choice=Paliperidone (Invega)), N (%) | 0 |  |  |  |  |  |
| Unchecked |  | 71 (100.0%) | 36 (100.0%) | 25 (100.0%) | 10 (100.0%) |  |
| Antipsychotics (choice=Asenapine (Saphris)), N (%) | 0 |  |  |  |  |  |
| Unchecked |  | 71 (100.0%) | 36 (100.0%) | 25 (100.0%) | 10 (100.0%) |  |
| Antipsychotics (choice=Iloperidone (Fanapt)), N (%) | 0 |  |  |  |  |  |
| Unchecked |  | 71 (100.0%) | 36 (100.0%) | 25 (100.0%) | 10 (100.0%) |  |
| Antipsychotics (choice=Brexpiprazole (Rexulti)), N (%) | 0 |  |  |  |  |  |
| Unchecked |  | 71 (100.0%) | 36 (100.0%) | 25 (100.0%) | 10 (100.0%) |  |
| Antipsychotics (choice=Cariprazine (Vraylar)), N (%) | 0 |  |  |  |  |  |
| Unchecked |  | 71 (100.0%) | 36 (100.0%) | 25 (100.0%) | 10 (100.0%) |  |
| Antipsychotics (choice=Haloperidol (Haldol)), N (%) | 0 |  |  |  |  |  |
| Unchecked |  | 71 (100.0%) | 36 (100.0%) | 25 (100.0%) | 10 (100.0%) |  |
| Antipsychotics (choice=Fluphenazine (Prolixin)), N (%) | 0 |  |  |  |  |  |
| Unchecked |  | 71 (100.0%) | 36 (100.0%) | 25 (100.0%) | 10 (100.0%) |  |
| Antipsychotics (choice=Chlorpromazine (Thorazine)), N (%) | 0 |  |  |  |  |  |
| Unchecked |  | 71 (100.0%) | 36 (100.0%) | 25 (100.0%) | 10 (100.0%) |  |
| Antipsychotics (choice=Thioridazine (Mellaril)), N (%) | 0 |  |  |  |  |  |
| Unchecked |  | 71 (100.0%) | 36 (100.0%) | 25 (100.0%) | 10 (100.0%) |  |
| Antipsychotics (choice=Perphenazine (Trilafon)), N (%) | 0 |  |  |  |  |  |
| Unchecked |  | 71 (100.0%) | 36 (100.0%) | 25 (100.0%) | 10 (100.0%) |  |
| Antipsychotics (choice=Molindone (Moban)), N (%) | 0 |  |  |  |  |  |
| Unchecked |  | 71 (100.0%) | 36 (100.0%) | 25 (100.0%) | 10 (100.0%) |  |
| Mood Stabilizers (choice=Lithium), N (%) | 0 |  |  |  |  |  |
| Unchecked |  | 71 (100.0%) | 36 (100.0%) | 25 (100.0%) | 10 (100.0%) |  |
| Mood Stabilizers (choice=Valproate (Depakote)), N (%) | 0 |  |  |  |  |  |
| Unchecked |  | 71 (100.0%) | 36 (100.0%) | 25 (100.0%) | 10 (100.0%) |  |
| Mood Stabilizers (choice=Lamotrigine (Lamictal)), N (%) | 0 |  |  |  |  |  |
| Unchecked |  | 71 (100.0%) | 36 (100.0%) | 25 (100.0%) | 10 (100.0%) |  |
| Mood Stabilizers (choice=Carbamazepine (Tegretol)), N (%) | 0 |  |  |  |  |  |
| Unchecked |  | 71 (100.0%) | 36 (100.0%) | 25 (100.0%) | 10 (100.0%) |  |
| Mood Stabilizers (choice=Oxcarbazepine (Trileptal)), N (%) | 0 |  |  |  |  |  |
| Unchecked |  | 71 (100.0%) | 36 (100.0%) | 25 (100.0%) | 10 (100.0%) |  |
| Mood Stabilizers (choice=Topiramate (Topamax)), N (%) | 0 |  |  |  |  |  |
| Unchecked |  | 71 (100.0%) | 36 (100.0%) | 25 (100.0%) | 10 (100.0%) |  |
| Mood Stabilizers (choice=Gabapentin (Neurontin)), N (%) | 0 |  |  |  |  |  |
| Unchecked |  | 71 (100.0%) | 36 (100.0%) | 25 (100.0%) | 10 (100.0%) |  |
| Mood Stabilizers (choice=Pregabalin (Lyrica)), N (%) | 0 |  |  |  |  |  |
| Unchecked |  | 71 (100.0%) | 36 (100.0%) | 25 (100.0%) | 10 (100.0%) |  |
| Mood Stabilizers (choice=Divalproex (Depakene)), N (%) | 0 |  |  |  |  |  |
| Unchecked |  | 71 (100.0%) | 36 (100.0%) | 25 (100.0%) | 10 (100.0%) |  |
| Anxiolytics (choice=Alprazolam (Xanax)), N (%) | 0 |  |  |  |  |  |
| Unchecked |  | 71 (100.0%) | 36 (100.0%) | 25 (100.0%) | 10 (100.0%) |  |
| Anxiolytics (choice=Clonazepam (Klonopin)), N (%) | 0 |  |  |  |  |  |
| Unchecked |  | 71 (100.0%) | 36 (100.0%) | 25 (100.0%) | 10 (100.0%) |  |
| Anxiolytics (choice=Lorazepam (Ativan)), N (%) | 0 |  |  |  |  |  |
| Unchecked |  | 71 (100.0%) | 36 (100.0%) | 25 (100.0%) | 10 (100.0%) |  |
| Anxiolytics (choice=Diazepam (Valium)), N (%) | 0 |  |  |  |  |  |
| Unchecked |  | 71 (100.0%) | 36 (100.0%) | 25 (100.0%) | 10 (100.0%) |  |
| Anxiolytics (choice=Chlordiazepoxide (Librium)), N (%) | 0 |  |  |  |  |  |
| Unchecked |  | 71 (100.0%) | 36 (100.0%) | 25 (100.0%) | 10 (100.0%) |  |
| Anxiolytics (choice=Temazepam (Restoril)), N (%) | 0 |  |  |  |  |  |
| Unchecked |  | 71 (100.0%) | 36 (100.0%) | 25 (100.0%) | 10 (100.0%) |  |
| Anxiolytics (choice=Buspirone (Buspar)), N (%) | 0 |  |  |  |  | 0.61 |
| Checked |  | 1 (1.4%) | 1 (2.8%) | 0 (0.0%) | 0 (0.0%) |  |
| Unchecked |  | 70 (98.6%) | 35 (97.2%) | 25 (100.0%) | 10 (100.0%) |  |
| Anxiolytics (choice=Hydroxyzine (Vistaril, Atarax)), N (%) | 0 |  |  |  |  |  |
| Unchecked |  | 71 (100.0%) | 36 (100.0%) | 25 (100.0%) | 10 (100.0%) |  |
| Talk Therapy, N (%) | 0 |  |  |  |  | **0.057** |
| No |  | 19 (26.8%) | 13 (36.1%) | 5 (20.0%) | 1 (10.0%) |  |
| Unknown |  | 43 (60.6%) | 16 (44.4%) | 18 (72.0%) | 9 (90.0%) |  |
| Yes |  | 9 (12.7%) | 7 (19.4%) | 2 (8.0%) | 0 (0.0%) |  |
| Duration of Therapy, N (%) | 70 |  |  |  |  |  |
| 3-6 months |  | 1 (100.0%) | 1 (100.0%) |  |  |  |
| Type of Birth, N (%) | 1 |  |  |  |  | 0.70 |
| Cesarean (c-section) |  | 17 (24.3%) | 10 (28.6%) | 5 (20.0%) | 2 (20.0%) |  |
| Vaginal |  | 53 (75.7%) | 25 (71.4%) | 20 (80.0%) | 8 (80.0%) |  |
| Reason for Cesarean Section, N (%) | 54 |  |  |  |  | 0.37 |
| 'Cat II tracing remote from delivery' |  | 1 (5.9%) | 0 (0.0%) | 0 (0.0%) | 1 (50.0%) |  |
| breech presentation and cHTN w/ SI PEC w/o SF |  | 1 (5.9%) | 1 (10.0%) | 0 (0.0%) | 0 (0.0%) |  |
| elective |  | 1 (5.9%) | 0 (0.0%) | 1 (20.0%) | 0 (0.0%) |  |
| fetal bradycardia |  | 1 (5.9%) | 1 (10.0%) | 0 (0.0%) | 0 (0.0%) |  |
| fetal malpresentation, severe preeclampsia with severe features |  | 1 (5.9%) | 1 (10.0%) | 0 (0.0%) | 0 (0.0%) |  |
| fetal malpresentation, twins |  | 1 (5.9%) | 1 (10.0%) | 0 (0.0%) | 0 (0.0%) |  |
| hsv |  | 1 (5.9%) | 0 (0.0%) | 1 (20.0%) | 0 (0.0%) |  |
| hx of c-section |  | 1 (5.9%) | 1 (10.0%) | 0 (0.0%) | 0 (0.0%) |  |
| hx of shoulder dystocia |  | 1 (5.9%) | 0 (0.0%) | 1 (20.0%) | 0 (0.0%) |  |
| hysterotomy |  | 1 (5.9%) | 0 (0.0%) | 1 (20.0%) | 0 (0.0%) |  |
| non-assuring FHTs |  | 1 (5.9%) | 1 (10.0%) | 0 (0.0%) | 0 (0.0%) |  |
| non-reassuring fhts |  | 1 (5.9%) | 1 (10.0%) | 0 (0.0%) | 0 (0.0%) |  |
| preeclampsia |  | 1 (5.9%) | 1 (10.0%) | 0 (0.0%) | 0 (0.0%) |  |
| previous C-sections |  | 1 (5.9%) | 1 (10.0%) | 0 (0.0%) | 0 (0.0%) |  |
| prior c-section |  | 1 (5.9%) | 0 (0.0%) | 0 (0.0%) | 1 (50.0%) |  |
| repeat |  | 1 (5.9%) | 1 (10.0%) | 0 (0.0%) | 0 (0.0%) |  |
| terminal fetal bradycardia |  | 1 (5.9%) | 0 (0.0%) | 1 (20.0%) | 0 (0.0%) |  |
| NICU admission? (Gestation Age < 35 weeks), N (%) | 1 |  |  |  |  | 0.48 |
| No |  | 67 (95.7%) | 34 (97.1%) | 23 (92.0%) | 10 (100.0%) |  |
| Yes |  | 3 (4.3%) | 1 (2.9%) | 2 (8.0%) | 0 (0.0%) |  |
| Postpartum Hemorrhage? (< /= 1000 mL at delivery), N (%) | 1 |  |  |  |  | 0.30 |
| No |  | 66 (94.3%) | 32 (91.4%) | 25 (100.0%) | 9 (90.0%) |  |
| Yes |  | 4 (5.7%) | 3 (8.6%) | 0 (0.0%) | 1 (10.0%) |  |
| Severe Preeclampsia?, N (%) | 1 |  |  |  |  | 0.26 |
| No |  | 61 (87.1%) | 31 (88.6%) | 20 (80.0%) | 10 (100.0%) |  |
| Yes |  | 9 (12.9%) | 4 (11.4%) | 5 (20.0%) | 0 (0.0%) |  |
| Complete?, N (%) | 0 |  |  |  |  | 0.61 |
| Complete |  | 70 (98.6%) | 35 (97.2%) | 25 (100.0%) | 10 (100.0%) |  |
| Incomplete |  | 1 (1.4%) | 1 (2.8%) | 0 (0.0%) | 0 (0.0%) |  |
| prenatal EPDS score1, N | 18 | 53 | 30 | 16 | 7 | **0.087** |
| Mean (SD) |  | 12.3 (6.4) | 12.7 (6.2) | 9.9 (6.9) | 16.1 (3.1) |  |
| Median (IQR) |  | 12.0 (8.0, 17.0) | 12.5 (10.0, 17.0) | 8.5 (6.0, 13.0) | 16.0 (14.0, 19.0) |  |
| Median (Range) |  | 12.0 (0.0, 25.0) | 12.5 (2.0, 25.0) | 8.5 (0.0, 23.0) | 16.0 (11.0, 20.0) |  |
| postpartum EPDS score1, N | 38 | 33 | 17 | 12 | 4 | 0.20 |
| Mean (SD) |  | 7.5 (5.5) | 7.1 (5.7) | 9.3 (5.6) | 3.8 (2.9) |  |
| Median (IQR) |  | 7.0 (3.0, 12.0) | 6.0 (2.0, 12.0) | 9.0 (6.5, 12.0) | 2.5 (2.0, 5.5) |  |
| Median (Range) |  | 7.0 (0.0, 19.0) | 6.0 (0.0, 17.0) | 9.0 (0.0, 19.0) | 2.5 (2.0, 8.0) |  |
| lost to follow up?, N (%) | 0 |  |  |  |  | 0.84 |
| incomplete |  | 1 (1.4%) | 1 (2.8%) | 0 (0.0%) | 0 (0.0%) |  |
| no |  | 34 (47.9%) | 18 (50.0%) | 12 (48.0%) | 4 (40.0%) |  |
| yes |  | 36 (50.7%) | 17 (47.2%) | 13 (52.0%) | 6 (60.0%) |  |
